# Generation of inactivated IL2RG and RAG1 monkeys with severe combined immunodeficiency using base editing

**DOI:** 10.1038/s41392-023-01544-y

**Published:** 2023-09-04

**Authors:** Xiao Zheng, Chunhui Huang, Yingqi Lin, Bofeng Han, Yizhi Chen, Caijuan Li, Jiawei Li, Yongyan Ding, Xichen Song, Wei Wang, Weien Liang, Jianhao Wu, Jiaxi Wu, Jiale Gao, Chengxi Wei, Xudong Zhang, Zhuchi Tu, Sen Yan

**Affiliations:** 1 Guangdong Key Laboratory of Non-human Primate Research, Guangdong-Hongkong-Macau Institute of CNS Regeneration, Jinan University, 510632 Guangzhou, China; 2 Department of Pathophysiology, School of Medicine, Jinan University, 510632 Guangzhou, China

**Keywords:** Genetic engineering, Experimental models of disease

## Abstract

Severe combined immunodeficiency (SCID) encompasses a range of inherited disorders that lead to a profound deterioration of the immune system. Among the pivotal genes associated with SCID, *RAG1* and *IL2RG* play crucial roles. *IL2RG* is essential for the development, differentiation, and functioning of T, B, and NK cells, while *RAG1* critically contributes to adaptive immunity by facilitating V(D)J recombination during the maturation of lymphocytes. Animal models carrying mutations in these genes exhibit notable deficiencies in their immune systems. Non-human primates (NHPs) are exceptionally well-suited models for biomedical research due to their genetic and physiological similarities to humans. Cytosine base editors (CBEs) serve as powerful tools for precisely and effectively modifying single-base mutations in the genome. Their successful implementation has been demonstrated in human cells, mice, and crop species. This study outlines the creation of an immunodeficient monkey model by deactivating both the *IL2RG* and *RAG1* genes using the CBE4max system. The base-edited monkeys exhibited a severely compromised immune system characterized by lymphopenia, atrophy of lymphoid organs, and a deficiency of mature T cells. Furthermore, these base-edited monkeys were capable of hosting and supporting the growth of human breast cancer cells, leading to tumor formation. In summary, we have successfully developed an immunodeficient monkey model with the ability to foster tumor growth using the CBE4max system. These immunodeficiency monkeys show tremendous potential as valuable tools for advancing biomedical and translational research.

## Introduction

Severe combined immunodeficiencies (SCIDs) encompass a group of inherited disorders that profoundly undermine the functionality of the immune system. This results in the absence or diminished presence of T and B cells derived from the thymus gland and bone marrow, leading to impairments in both cellular and humoral adaptive immunity.^1^ The pathogenesis of SCID primarily involves the premature demise of precursor lymphocyte cells due to defects in purine metabolism, abnormalities in signaling through common γ chain-dependent cytokine receptors, anomalies in V(D)J recombination, and deficiencies in pre-TCR/TCR signaling.^2^ Several gene mutations have been identified as contributors to SCIDs, including *MALT1*, *ZAP70*, *IL21R*, *FOXN1*, *CORO1A*, *RAG1/2*, *IL2RG*, *AK2*, *IKBK2*, *UNC119*, *LCK*, *TTC7A*, *CARD11*, and *BCL10*.^1,3,4^ Among these, IL2RG, also known as the common gamma chain, acts as a subunit of the IL-2 receptor shared by IL-21, IL-15, IL-9, IL-4, and IL-7 receptors.^5,6^ Mutations in *IL2RG* are responsible for X-linked severe combined immunodeficiency (X-SCID), which constitutes the most prevalent form of SCID, accounting for 50% of all cases. In X-SCID, T and NK cells are either absent or significantly diminished, while B cells exhibit functional impairments, with their numbers being normal or increased.^1,7^ Another gene associated with SCID is *RAG1*, which plays a crucial role in adaptive immunity by facilitating V(D)J recombination in developing lymphocytes.^3^ The RAG recombinase (RAG1/2) randomly combines variable (V), diversity (D), and joining (J) gene segments to generate a V(D)J exon, which encodes the variable region of T cell receptors and antibodies. Numerous loss-of-function mutants of RAG1 have been identified as the causative factors of SCID in humans.^8,9^ These mutants are characterized by partial protein expression and limited production of T and B cells while retaining normal NK cell functionality.^10^

Animals with impaired immune systems can be categorized as either genetically immunodeficient due to congenital mutations or artificially induced through biotechnological methods. Currently, there are several types of genetically immune-deficient animal models, such as mice, rats, and pigs.^7,11–13^ These immunodeficient animals have a wide range of applications in fields including oncology, stem cell therapy, immune system research, and infectious diseases.^12,13^ Humanized small animal models are particularly valuable in immuno-oncology research, as they offer potential for clinical translation. However, the accurate selection of appropriate models for specific situations remains challenging. It is currently impossible to find a mouse model that fully replicates all aspects of human biology, and some SCID mice develop immune leaks as they age. These leaks result in the production of small numbers of functional T and B cells, as well as immunoglobulins, in mice. The molecular mechanism behind immune leakage in SCID mice remains unclear, and there are currently no established diagnostic criteria. Therefore, it becomes crucial to establish an immunodeficiency model that closely mimics human biology in order to address the limitations of animal model research. Non-human primates (NHPs) are highly phylogenetically related to humans and share many physiological similarities, including immune system characteristics.^14^ The close genetic and physiological resemblance between NHPs and humans makes them excellent models for biomedical research. Consequently, the establishment of an immunodeficiency monkey model becomes imperative.

While CRISPR/Cas9 holds promise for therapeutic and biological research, concerns arise due to its reliance on DNA double-strand breaks (DSBs) to facilitate gene editing. CRISPR/Cas9-induced DSBs can result in the deletion of thousands of base pairs, leading to the generation of genotypes that may have detrimental effects on mitotically active cells. For instance, when applied to human pluripotent stem cells, CRISPR/Cas9 can induce p53 mutations, limiting the feasibility of cell replacement therapy. Additionally, the efficiency of gene editing is impeded by high frequencies of insertion-deletion mutations, with CRISPR-mediated homology-directed repair rates in HEK293T cells reaching only 38%. However, the majority of known genetic diseases are primarily caused by single-nucleotide polymorphisms (SNPs). Consequently, there is a demand for methods that can specifically modify individual base pairs at the target site without introducing DSBs. Base editors provide programmability and flexibility, eliminating the need for DSBs and overcoming the limitations of traditional Cas9 nucleases in gene editing.^15–19^ Cytosine base editors (CBEs), which possess cytosine deaminase activity, have been effectively utilized to introduce precise single-base mutations in the genomes of various organisms, including humans, mice, and crops.^20–22^ The CBE4max system, an enhanced tool for C-G to T-A conversion compared to CBE4, has been developed and validated in a mouse model and subsequently optimized for mammalian species.^23,24^

This study successfully achieved the generation of an immunodeficiency model in non-human primates by utilizing the CBE4max system to introduce specific single-nucleotide substitutions in the *IL2RG* and *RAG1* genes. The base editing procedure resulted in a notable impairment of the immune system in the edited monkeys, manifesting as lymphopenia, atrophy of lymphoid organs, and an absence of mature T cells. Furthermore, we observed the remarkable ability of human breast cancer cells to survive and proliferate within the base-edited monkeys. These findings serve as compelling evidence for the effectiveness of our methodology in establishing a robust immunodeficiency monkey model, which has tremendous potential for investigating tumor biology and facilitating advancements in biomedical and translational research. To conclude, the immunodeficiency monkeys generated in this study offer promising prospects for future medical investigations and breakthroughs.

## Results

### Generation of base-edited monkey model

**Fig. 1:**
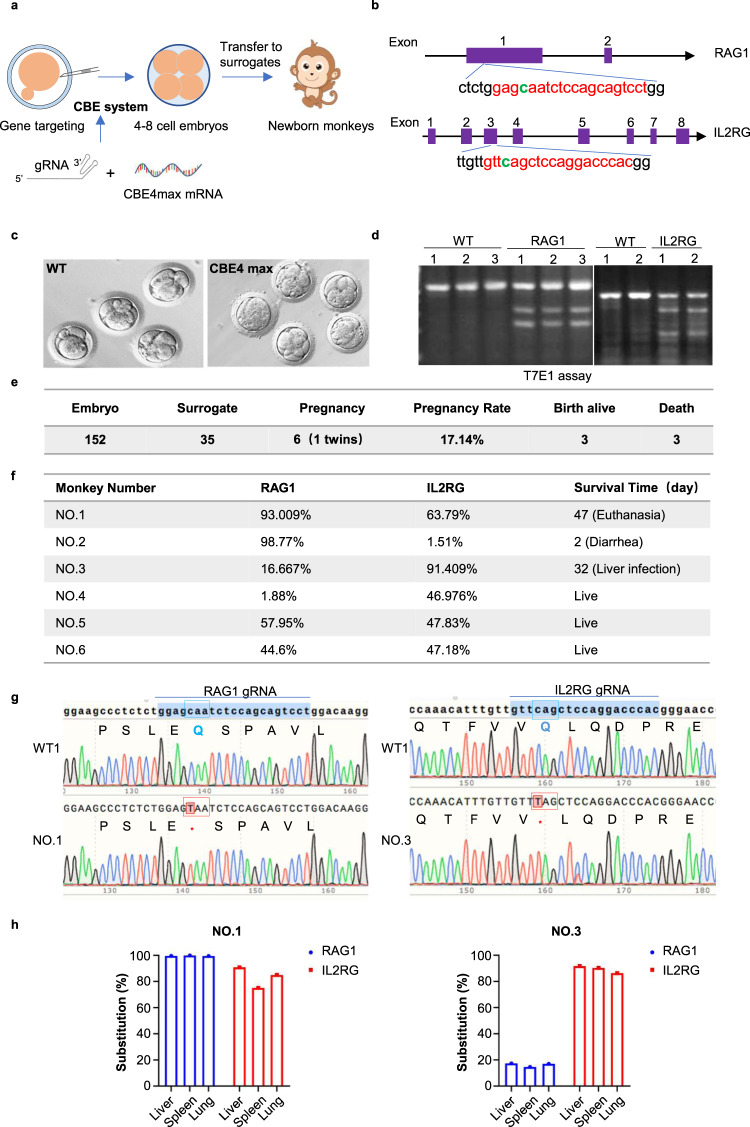
Base-edited monkeys were generated using the CBE4max system. **a** Schematic illustrating the process of generating base-edited monkeys. **b** Specific gRNAs designed to target *RAG1* and *IL2RG* genes. The gRNA target sites are highlighted in red, while the edited bases are indicated in green. **c** The status of the embryos after CBE4max systemic injection. **d** T7E1 assay for base-edited embryos. **e** Following the microinjection of 152 embryos, 35 embryos in the developmental stage were transferred to surrogate female monkeys. Subsequently, five recipient monkeys were confirmed to be pregnant, resulting in the successful acquisition of six newborn monkeys. **f** The editing efficiency in the peripheral blood of the edited animals was assessed using Sanger sequencing-based EditR software. **g** The genotype of the base-edited monkeys (mutation) was determined. Red boxes indicate the presence of stop codons after the substitution, while the asterisk (*) denotes a substituted base. The gRNA sequencing map of the *RAG1* gene is derived from monkey NO.1, while the gRNA sequencing map of the *IL2RG* gene is from monkey NO.3. **h** The frequencies of base substitution in deceased base-edited monkeys determined through targeted deep sequencing

To establish monkeys with mutations in the *RAG1* and *IL2RG* genes, we employed gene editing techniques in monkey embryos, following the procedures described in our previous studies.^25,26^ Specifically, we designed guide RNAs (gRNAs) targeting exon 1 of *RAG1* and exon 3 of *IL2RG* (Fig. 1a, b) and co-injected CBE4max mRNA and gRNAs into fertilized embryos of cynomolgus monkeys (*Macaca fascicularis*). Prior to injection, we evaluated gRNA activity in monkey embryos using the T7E1 method. The T7E1 enzyme digestion confirmed effective mutations in the target genes within the base-edited embryos (Fig. 1c, d). To validate the genotype of the edited embryos, we performed Sanger sequencing (Supplementary Fig. 1). A total of 152 embryos at the 4–8 cell stage were transferred to 35 surrogates, resulting in five pregnancies that led to the birth of six newborn monkeys (Fig. 1e). Genomic DNA was extracted from blood samples of the newborn monkeys, and PCR-based Sanger sequencing confirmed the presence of edits at the target sites in all six monkeys (Fig. 1f). Sanger sequencing was also utilized to verify the genotypes of the edited animals, revealing C to T nucleotide substitutions at the mutated sites. These substitutions introduced stop codons in the corresponding genomic gRNA sequences (Fig. 1g). Unfortunately, two out of the six edited monkeys succumbed to diseases: diarrhea (NO.2) and liver infection (NO.3), while one edited monkey was euthanized for tumor sample collection. Additionally, we extracted genomes from the spleen, lung, heart, and liver of two monkeys (NO.1 and NO.3) and examined the target regions using PCR-based Sanger sequencing. Variations in editing efficiency were observed among different tissues in the two monkeys (Supplementary Fig. 2a–c). Furthermore, we assessed the base editing efficiency of the *RAG1* and *IL2RG* genes in the tissues (spleen, lung, and liver) of the deceased monkeys and in the peripheral blood of the surviving edited monkeys using targeted deep sequencing (Fig. 1h and Supplementary Fig. 2d). The NO.1 monkey exhibited higher base editing efficiency for both the *RAG1* and *IL2RG* genes, whereas the NO.3 monkey displayed significantly higher base editing efficiency for the *IL2RG* gene compared to *RAG1*. In conclusion, we have successfully generated monkeys with mutations in the *RAG1* and *IL2RG* genes using the CBE4max system.

### Phenotypes of base-edited monkeys

**Fig. 2:**
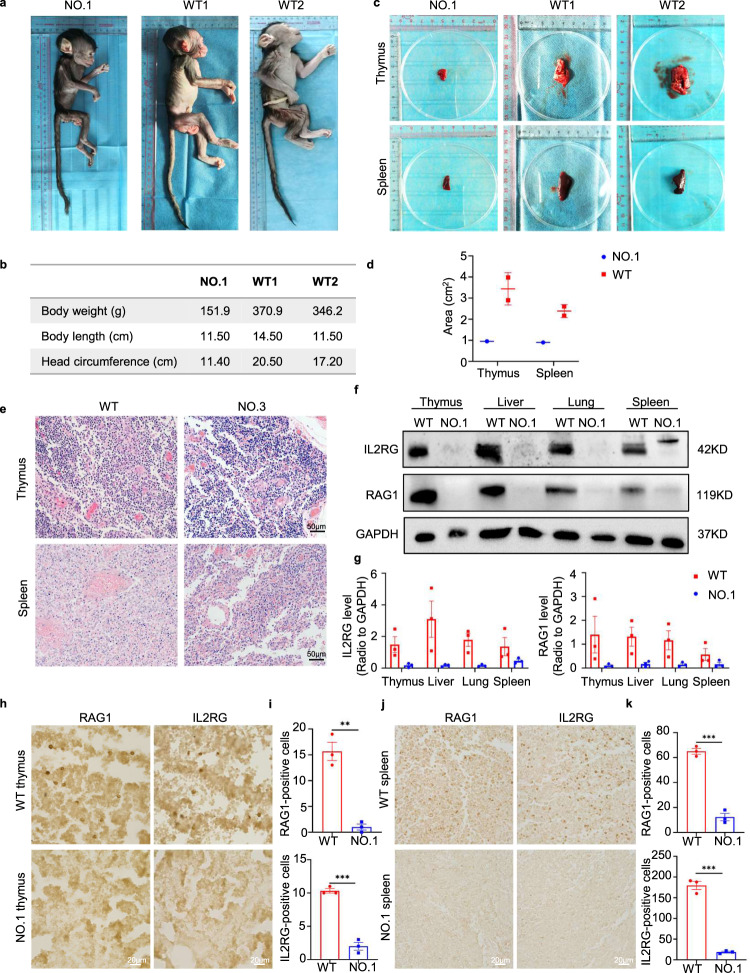
Phenotypic and histopathological changes in base-edited monkeys. **a**, **b** Comparison of body weights, body lengths, and head circumferences between mutant monkeys and age-matched WT monkeys while being subjected to the same feeding environment. **c**, **d** Analysis of thymus and spleen in base-edited monkeys. **e** H&E staining depicting the development of thymus and spleen in base-edited monkeys. Scale bars: 50 µm. **f**–**k** Evaluation of RAG1 and IL2RG protein expression in the thymus and spleen of mutant monkeys. Western blot analysis illustrating RAG1 and IL2RG protein expression levels and quantification in the thymus, spleen, liver, and lung of mutant and WT monkeys (**f**, **g**). Immunohistochemistry depicting the expression and quantification of RAG1 and IL2RG in the thymus and spleen of mutant and WT monkeys (**h**–**k**). Statistical analysis was performed using an unpaired two-tailed *t*-test to compare the two groups. *P* < 0.05 was considered statistically significant. Three replicates were performed for each analysis. Scale bars: 20 µm

Prior investigations have provided evidence of the impact of *RAG1* and *IL2RG* gene mutations on the physical characteristics and histopathological features of edited animals.^27^ Therefore, we conducted a comprehensive analysis comparing the physical attributes of base-edited monkeys to control monkeys. Notably, the base-edited monkeys exhibited significantly lower body weights, body lengths, and head circumferences compared to age- and gender-matched wild-type (WT) monkeys (Fig. 2a, b). Additionally, post-mortem examinations were conducted on both the base-edited monkeys and WT monkeys. The results unveiled reduced sizes of the thymus and spleen in the base-edited monkeys when compared to the WT monkeys (Fig. 2c, d). To gain further insights into the internal structure and cellular composition, we performed H&E staining on the thymus and spleen. Abnormalities in development were evident in both organs of the mutant monkeys, including thymic lobule atrophy. In the spleen of the mutant monkeys, the periarterial lymph sheath surrounding the central artery was hypoplastic, and the tissue exhibited notable looseness (Fig. 2e). Furthermore, we assessed the expression levels of RAG1 and IL2RG proteins in the thymus, spleen, liver, and lung of the base-edited monkeys. Western blot analysis revealed a significant decrease in the expression levels of RAG1 and IL2RG in these tissues (Fig. 2f, g). Immunohistochemical staining further confirmed a marked reduction in the expression of RAG1 and IL2RG in the thymus and spleen (Fig. 2h–k).

### Immune system changes in base-edited monkeys

**Fig. 3:**
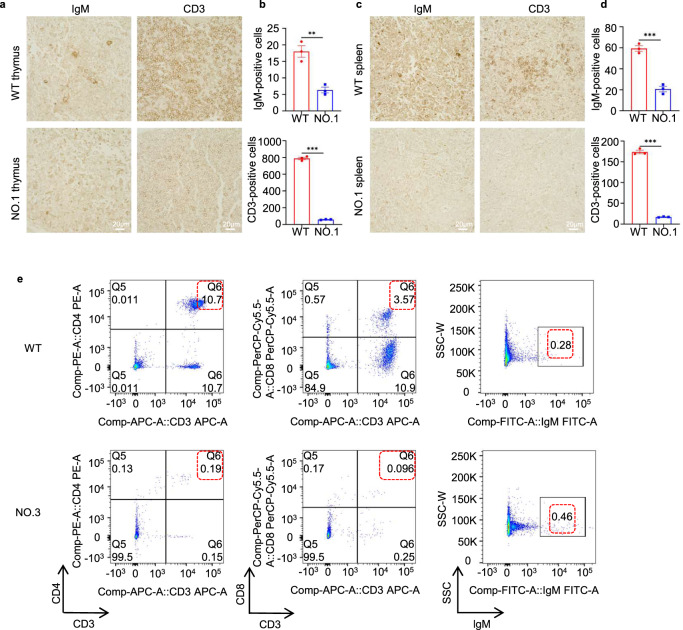
Status of T and B lymphocytes in base-edited monkeys. **a**–**d** Immunohistochemistry was performed to assess the presence of IgM^+^ and CD3^+^ cells in the thymus (**a**, **b**) and spleen (**c**, **d**). Quantitative analysis was conducted on WT and mutant monkeys. Statistical analysis of the data was carried out using an unpaired two-tailed *t*-test to compare the two groups. A significance level of *P* < 0.05 was considered statistically significant. Each experiment was repeated three times. Scale bars: 20 µm. **e** Representative flow cytometry results of peripheral blood lymphocytes in WT and mutant monkeys

To evaluate the status of T lymphocytes and B lymphocytes in the base-edited monkeys, we conducted immunohistochemical staining of the thymus and spleen, as well as analyzed T and B lymphocytes in peripheral blood. Initially, we performed immunohistochemical staining using IgM as a B lymphocyte marker and CD3 as a T lymphocyte marker. The findings revealed a reduction in IgM and CD3 expression in the thymus and spleen of mutant monkeys compared to WT monkeys (Fig. 3a–d). Additionally, we collected peripheral blood mononuclear cells and analyzed T and B cell expression through flow cytometry. After staining T lymphocytes with CD3, CD4, and CD8, we observed a significant decrease in the ratio of CD3^+^CD4^+^ T lymphocytes to CD3^+^CD8^+^ T lymphocytes in the mutant monkeys (<1%) compared to the percentages of 10.7% and 3.57% observed in WT monkeys, respectively (Fig. 3e). However, no notable changes were observed in the population of IgM^+^ B cells in the blood of the mutant monkeys compared to the WT monkeys. In addition to T and B cells, NK cells are an essential type of immune cell derived from the bone marrow and represent the third largest subset of lymphocytes, alongside T cells and B cells. They primarily reside in the spleen, peripheral blood, and liver. Since *IL2RG* plays a crucial role in the development, differentiation, and function of T, B, and NK cells, defects in this gene lead to severe immunodeficiency. Therefore, we performed immunohistochemical staining of the spleens from the base-edited monkeys. The results demonstrated a significant decrease in the expressions of CD56 and CD16 in the spleens of base-edited monkeys NO.1 and NO.3 (Supplementary Fig. 3).

### Off-target analysis

**Fig. 4:**
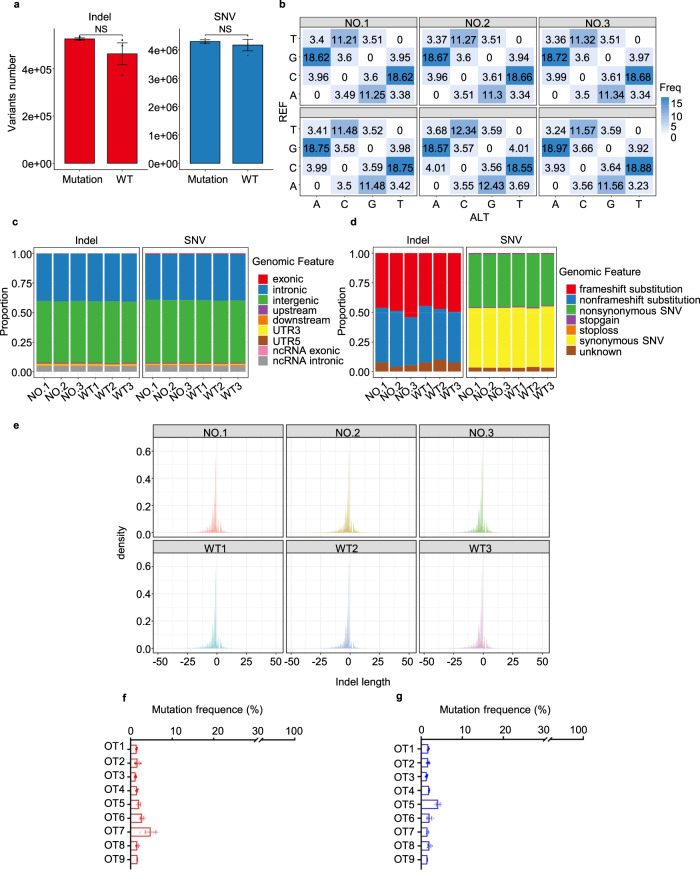
Off-target analysis in base-edited monkeys. **a** Whole-genome sequencing analysis was conducted to identify single-nucleotide variations (SNVs) and indels in three base-edited monkeys (NO.1, NO.2, and NO.3) as well as three wild-type monkeys (WT1, WT2, and WT3). The height of the bars represents the average number of mutations in each group, while black dots indicate the number of mutations in each sample. **b** The distribution of mutation types in base-edited and wild-type monkeys. The number within each cell indicates the proportion of a specific mutation type among all mutations. **c** The genomic feature regions of mutations in base-edited and wild-type monkeys. A large proportion of SNVs were detected in intronic and intergenic regions, and no difference was observed between base-edited and wild-type monkeys. **d** Evaluation of the potential for mutations in base-edited and wild-type monkeys to impact gene function. No difference was observed between base-edited and wild-type monkeys. **e** Distribution of indel fragment lengths. **f**, **g** Assessment of Off-target base editing frequency in the *RAG1* (**f**) and *IL2RG* (**g**) genes in base-edited monkeys generated by the CBE4max system. Potential off-target sites (OT1–OT9) were detected by deep sequencing and predicted using Cas-OFFinder. Deep sequencing was employed to determine the frequency of substitutions at predicted target sites in the spleen (NO.1 and NO.3) and peripheral blood (NO.2) of three deceased base-edited animals

To examine any potential off-target effects of the base-edited monkeys, we conducted whole-genome sequencing (WGS) on the genomes of three base-edited monkeys (NO.1, NO.2, and NO.3) and three wild-type monkeys (WT1, WT2, and WT3). Initially, we compared the number of single-nucleotide variations (SNVs) and indels between the base-edited and wild-type monkeys. The results showed no significant difference in the number of SNV and indel mutations between the two groups (Fig. 4a). Subsequently, we analyzed whether these SNV mutations exhibited any preferences for specific bases and found that the distribution of base mutations in the mutant monkeys was similar to that in the wild-type monkeys (Fig. 4b). We did not observe any notable disparities in genome feature regions or changes in gene function caused by mutations between the base-edited and wild-type monkeys (Fig. 4c, d). Moreover, there were no significant deviations in the distribution of indel fragment lengths (Fig. 4e). Furthermore, we assessed potential Cas9-dependent off-target effects by designing nine potential off-target sites for the *IL2RG* and *RAG1* genes using Cas-OFFinder. We used deep sequencing to predict the off-target efficiency at these sites in the genomes of the spleen (NO.1 and NO.3) and peripheral blood (NO.2) of the three deceased edited animals (Fig. 4f, g and Supplementary Fig. 4). Our data demonstrated that editing *IL2RG* and *RAG1* using the CBE4max system did not result in significant off-target mutations.

### Base-edited monkeys support the growth of tumor cells

**Fig. 5:**
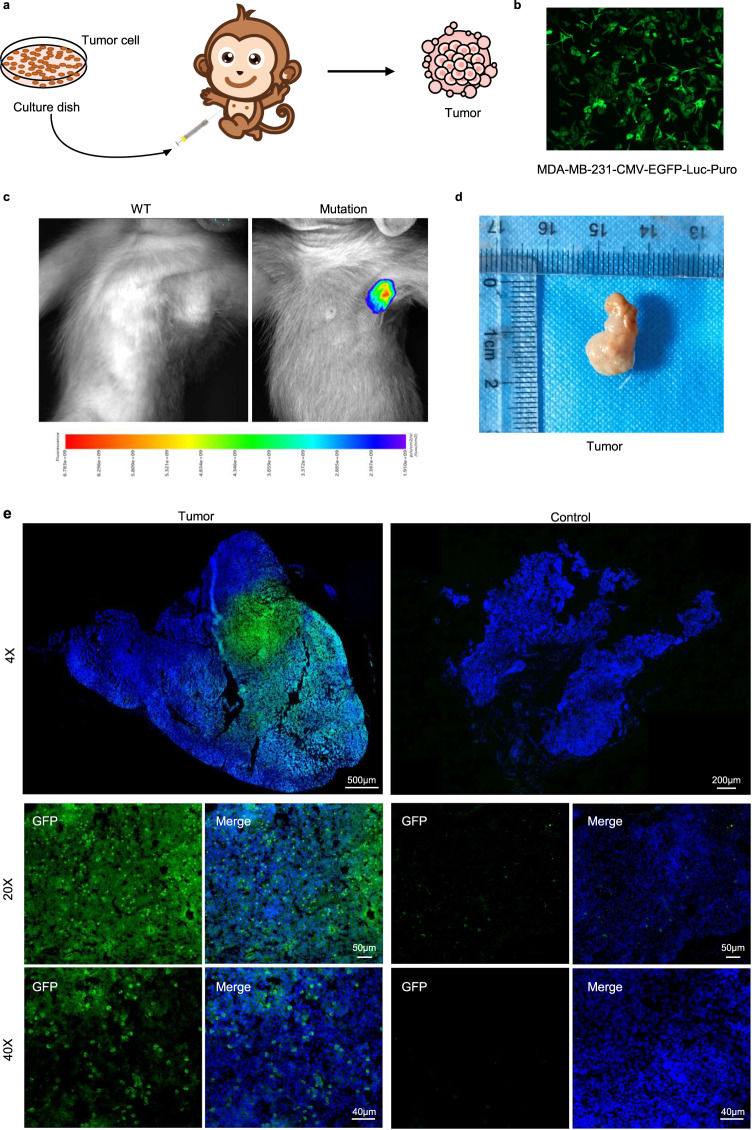
Base-edited monkeys support tumor growth. **a** Schematic diagram illustrating the growth of human breast cancer cells in base-edited monkeys. **b** Fluorescence microscopy image displaying the GFP signal emitted by human breast cancer cells. **c** In vivo imaging of animals to observe the status of tumor. **d** Examination of tumor tissues in base-edited monkeys. **e** Immunofluorescence staining of tumor tissues indicating extensive expression of GFP protein. Scale bars: 4X: Tumor = 500 µm Control = 200 µm, 20X = 50 µm, 40X = 40 µm

**Fig. 6:**
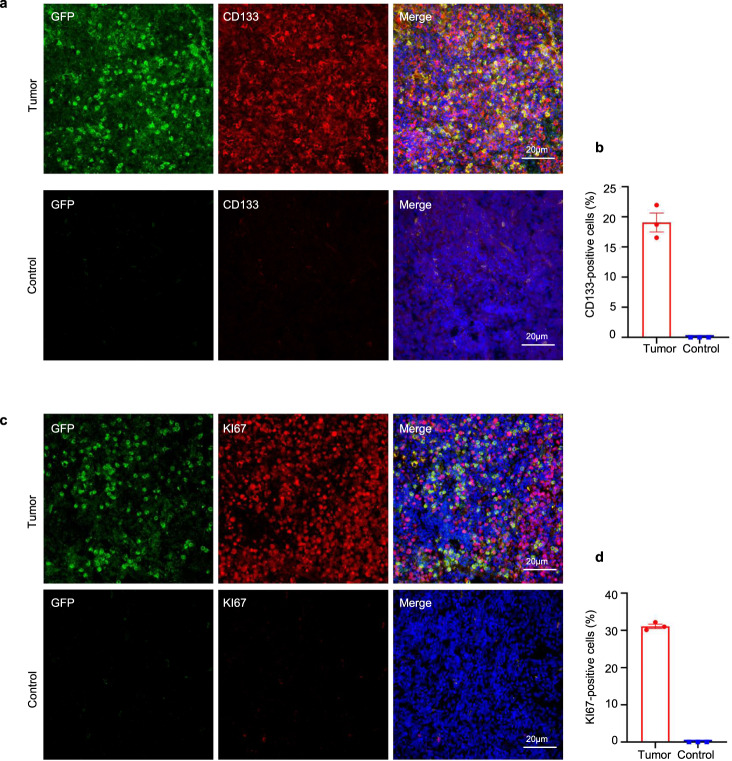
Validation of tumor tissues. **a**–**d** Tumor tissues were labeled with KI67 and CD133 markers to assess tumor growth. Statistical analysis of the data was performed using an unpaired two-tailed *t*-test to compare the two groups. A significance level of *P* < 0.05 was considered statistically significant. Each experiment was repeated three times. Scale bars: 20 µm

**Fig. 7:**
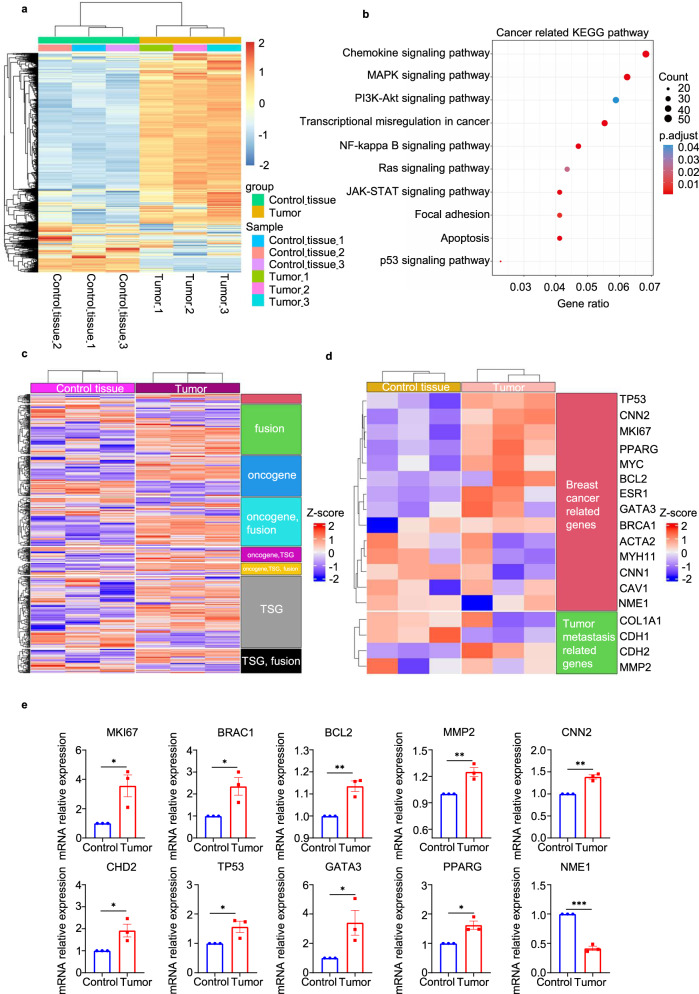
RNA-seq analysis of tumor tissues. **a** Heat map illustrating the differentially expressed genes between the tumor group and the control tissue group (log2FC > 1; *P*_adj_ < 0.01). **b** KEGG gene set enrichment analysis based on RNA-Seq data by comparing tumor groups with control tissue groups to identify alterations in tumor-associated signaling pathways (*P*_adj_ < 0.05). **c** Heat map showcasing the screening of the tumor-related gene list provided by the COSMIC Cancer Gene Census. The classification is based on the “Role in Cancer” information provided by the COSMIC database. **d** Heat map displaying the expression of genes related to breast cancer. **e** Validation of the expression levels of tumor-associated genes using RT-qPCR. Statistical analysis of the data was conducted using an unpaired two-tailed *t*-test to compare the two groups. A significance level of *P* < 0.05 was considered statistically significant

To assess the feasibility of using base-edited monkeys as an in vivo model for studying human oncology, we introduced human breast cancer cells (MDA-MB-231-CMV-EGFP-Luc-Puro) into both mutant and WT monkeys (Fig. 5a). Subcutaneous inoculation of 1 × 10^7^ cells into the axillae of the monkeys was performed, followed by in vivo tumor imaging after 3 weeks. The images clearly demonstrated the presence of signals indicating tumor cell growth (Fig. 5b, c and Supplementary Fig. 5). Upon euthanizing the mutant monkeys, we collected the transplanted human tumors and subjected them to staining for examination (Fig. 5d). Immunofluorescence analysis revealed the presence of GFP signals originating from the injected tumor cells in the harvested tumors, indicating the inability of the mutant monkeys to reject solid human tumors (Fig. 5e). Furthermore, we conducted staining for KI67 and CD133, widely expressed markers in tumor tissues, further confirming robust tumor growth in the base-edited monkeys (Fig. 6a–d). To gain insights into the genetic characteristics of the tumor and control groups, we performed RNA-seq analysis to determine global gene expression patterns. Total RNA was extracted from the samples for transcriptome analysis. Comparative analysis between the tumor and control groups revealed 3151 differentially expressed genes, including 1663 upregulated genes and 1488 down-regulated genes, in the tumor group (Fig. 7a). KEGG enrichment analysis of these differentially expressed genes indicated their enrichment in tumor-related signaling pathways (*P*_adj_ < 0.05) (Fig. 7b). Moreover, when comparing the list of tumor-related genes provided by the COSMIC Cancer Gene Census, we observed significant differences in gene expression between the tumor and control groups (Fig. 7c and Supplementary Fig. 6). Additionally, we screened 18 breast cancer-related genes and found that the expression of tumor-related genes supported tumor growth (Fig. 7d). To validate the RNA-seq analysis, we selected 10 genes for RT-qPCR and confirmed that their expression patterns were consistent with the RNA-seq results (Fig. 7e). These findings strongly support the use of mutant monkeys as a viable in vivo model for studying human oncology.

## Discussion

In this study, we utilized the CBE4max system to generate an immunodeficient monkey model by disabling the *IL2RG* and *RAG1* genes. However, precise genetic modifications using CRISPR-Cas9 rely on the HDR pathway, which involves DSBs and external donor DNA templates. Unfortunately, CRISPR-Cas9-induced DSBs can result in various genomic rearrangements, including large chromosomal deletions, inversions, translocations, and even catastrophic events like chromothripsis, aneuploidy, chromosome loss, p53 activation, and integration of exogenous sequences such as lentivirus, adeno-associated virus (AAV), LINE-1 retrotransposons, and plasmids.^28^ Moreover, HDR is a rare event (typically ~0.1–5%), especially in non-dividing cells, which poses challenges for precise genome editing in several species. On the other hand, base editing provides a highly efficient and accurate approach for generating targeted single-nucleotide mutations. Unlike CRISPR-Cas9, base editing does not involve cleaving the nucleic acid backbone. Instead, it chemically modifies the target nucleotide during genome and transcriptome editing. Specifically, CBEs enable the creation of precise point mutations without DSBs or the need for donor DNA templates, resulting in enhanced editing efficiency.^29^

In a related study focusing on the construction of an immunodeficient monkey model, Niu et al. were the first to employ the CRISPR/Cas9 system to target the monkey genome. They successfully achieved precise gene targeting of *Ppar-γ* and *Rag1* in rhesus monkeys by co-injecting Cas9 mRNA and sgRNA into single-cell stage embryos. However, the phenotype of the Rag1 knockout monkey was not reported in their study.^30^ Although immunodeficient animal models such as mice,^31^ rats,^32^ and rabbits^33^ have been generated through *IL2RG* and *RAG1* mutations, our study is the first to demonstrate that mutations in these two genes yield an immunodeficient phenotype in monkeys. While invaluable insights into basic biological processes have been derived from research involving small animal models, their limited ability to accurately replicate human biological systems, particularly the immune system, hampers the study of human biology.^34^ During the development of anticancer drugs mediated by STING agonists, researchers observed that human and rat STING exhibited more similar signaling profiles with DMXAA and CMA than human and mouse STING, highlighting species-specific differences that provide ideas for constructing immunodeficient monkey models.^34,35^ Monkeys share greater physiological structural and functional similarities with humans, underscoring the significance of utilizing monkeys in constructing immunodeficient models. The *IL2RG* and *RAG1*-deficient monkeys described in this study hold value not only for oncology research but also represent a significant step forward in utilizing monkeys as models for severe immunodeficiency diseases in humans and regenerative medicine.

Furthermore, the issue of off-target effects is always a significant concern in gene editing.^29^ To thoroughly investigate the presence of off-target effects, we employed targeted deep sequencing to detect 18 potential off-target sites (9 for *IL2RG*-sgRNA and 9 for *RAG1*-sgRNA). To validate these findings, PCR followed by Sanger sequencing was conducted, confirming 24 off-target sites (12 for *RAG1*-sgRNA and 12 for *IL2RG*-sgRNA). Additionally, whole-genome sequencing was utilized to identify any Cas9-independent off-target effects. Encouragingly, the results indicated no significant off-target mutations.

Moreover, we observed that the base-edited monkeys exhibited significantly smaller body sizes and immune organ volumes compared to age-matched WT monkeys. Additionally, the mutant monkeys displayed reduced numbers of mature lymphocytes in their blood, spleen, and thymus, which play a crucial role in allogeneic rejection. Notably, no significant changes in the number of B cells in peripheral blood were observed. These pathological changes closely align with the expected phenotype of an immunodeficient model. While immunodeficient mouse models have shown the ability to support the proliferation of primary human tumors,^36,37^ their limited capacity to predict responses to anti-human cancer drugs stems from substantial physiological differences between rodents and humans.^38^ In contrast, the base-edited monkey model presents a promising preclinical testing platform to explore its potential in supporting human tumor growth and developing specific and effective cancer treatment drugs. Interestingly, after implanting tumor cells, we observed the emergence of tumor tissues in the mutant monkeys. Immunohistological analysis and in vivo imaging confirmed that these tumor tissues originated from the injected tumor cells. Furthermore, the presence of cells exhibiting distinct tumor stem cell properties, such as CD133, was detected. Additionally, RNA-seq analysis provided further confirmation of the existence of human tumor tissues.

Overcoming the limited availability of immunodeficient monkeys for research is a significant challenge due to their long generation cycle. Therefore, it is crucial to explore methods that can yield a larger number of genetically edited monkeys. One promising solution is to accelerate sperm maturation by xenotransplanting testicular tissue, which can reduce the time required for the generation cycle. By combining this approach with efficient gene editing techniques, non-human primates, such as monkeys, become invaluable animal models for both basic and biomedical research.^39,40^ Another approach involves utilizing gene-edited donor somatic cells for somatic cell nuclear transfer (SCNT). This method allows for the generation of a genetically consistent set of gene-modified animals without chimerism or hybrid breeding. Thus, the SCNT-based approach holds significant potential for generating gene-modified monkey models.^41,42^ These methods offer promising avenues to expedite the production of base-edited monkeys, enabling researchers to acquire a greater number of monkey models in the future.

In summary, we have successfully generated *IL2RG* and *RAG1* mutant monkeys with severe combined immunodeficiency using the CBE4max base editing system. These immunodeficient monkeys serve as valuable tools for preclinical research and bridge the gap between small animal models and humans. Their utilization can significantly enhance the efficacy of preclinical studies for the development of anticancer agents and the transplantation of heterologous cells or organs in the field of regenerative medicine.

## Materials and methods

### Animals

The gene targeting experiment was conducted on cynomolgus monkeys, which served as the experimental subjects. These monkeys were housed at Guangdong LANDAU Biotechnology Co. Ltd and TOPGENE Biotechnology Co. Ltd, where they received unrestricted access to water and were provided with a standard diet in accordance with standard care practices for monkeys. The experimental protocol adhered to the Guidelines for the Care and Use of Laboratory Animals established by Jinan University. Approval for this experiment was granted by the Animal Care and Use Committee of LANDAU (approval number: LDACU20201224-01).

### Vector construction

The SgRNA sequences targeting the *RAG1* and *IL2RG* genes were designed using online software (see detailed sequences in Fig. 1b). These oligonucleotides were then annealed and inserted into pBluescriptSKII-U6-sgRNA expression vectors for cloning purposes.

### mRNA and RNA preparation

The CBE4max plasmids were acquired from Addgene and subsequently linearized using Not I. In order to synthesize mRNA, in vitro RNA transcription was performed using the HiScribe™ T7 ARCA mRNA Kit (tailed) from NEB. The SgRNAs were amplified and transcribed in vitro utilizing the MAXIscript T7 kit (Ambion). Following transcription, purification of the SgRNAs was carried out using the miRNeasy Mini Kit (Qiagen), following the instructions provided by the manufacturer.

### Monkey zygote microinjection and animal breeding

A combination of CBE4max mRNA (200 ng/μL) and sgRNA (50 ng/μL) was injected into the zygotes of monkeys using microinjection techniques. The reconstructed embryos were subsequently placed in a culture medium for further development. Once they reached the 4–8 embryonic stage, the embryos were surgically transferred into the fallopian tubes of surrogate monkeys. Following birth, genotyping was conducted on the offspring to validate the successful breeding of animals.

### PCR analysis to sequence

Tissue samples were collected from the mutant monkeys, and DNA extraction was carried out to obtain genomic DNA. For PCR analysis, rTaq polymerase (Takara, Kyoto, Japan) was used with the following cycling parameters: initial denaturation at 94°C for 3 min, followed by 30 cycles of denaturation at 94°C for 30 s, annealing at 65°C for 30 s, and extension at 72°C for 5 min. A final extension step was performed at 72°C for 4 min. The genotyping primers used were as follows: *IL2RG* (forward 5′-GGCTCTGGATGACTGCGGTACC-3′; reverse 5′-GTCAGTCCGGTACTGCACCAAGTG-3′); *RAG1* (forward 5′-TCAGCCAGCATGGCGTCCTCTTT-3′; reverse 5′-GAACTGGATATCTCCTGTTGTGCTCA-3′). Subsequently, Sanger sequencing analysis was conducted on the PCR products to examine the genetic sequences.

### T7E1 assay

To identify specific DNA mutations in the PCR products, we employed the T7E1 assay. The PCR products were denatured and then re-annealed in NEBuffer 2 (NEB) using a thermal cycler. After re-annealing, the PCR products underwent digestion using T7 endonuclease 1 (NEB, M0302L) and were subsequently separated through agarose gel electrophoresis for analysis.

### Whole-genome sequencing

GuangZhou HeQin Bio Tec conducted the preparation of monkey DNA libraries and performed whole-genome sequencing (WGS) using the Illumina NovaSeq 6000 platform. The WGS achieved an average coverage of 30×. To process the raw sequencing data, fastp (v0.20.1) was utilized to eliminate low-quality reads and trim adapter sequences. The remaining high-quality sequencing reads were then aligned to the reference genome (Macaca_fascicularis_6.0) using BWA (v0.7.15-r1140). The resulting BAM files underwent additional processing, including read sorting, duplicate removal, and BAM file indexing, using sambamba (v0.6.6). For germline variant detection, variant calling was performed using strelka (v2.9.10). The raw variants generated by strelka, which had a filter tag of “PASS” were considered high-confidence variants for subsequent analysis. To annotate single-nucleotide variants (SNVs) and insertions/deletions (indels), ANNOVAR (v2017Jul17) was employed for genomic annotation. Custom R scripts were used for downstream analysis and visualization purposes.

### Estimated edit frequency

To assess the efficiency of base editing using Sanger sequencing, we employed the EditR online software (https://moriaritylab.shinyapps.io/editr_v10/) as a tool for estimating the editing frequency.^43^

### Histopathological analysis and immunohistochemistry

The excised tissue was immersed in 4% paraformaldehyde for 3 days to initiate fixation. Subsequently, the tissues were embedded in paraffin and sectioned at a thickness of 3 μm for both H&E staining and immunofluorescence (IF) analysis. For IF staining, antigen retrieval solution (Sigma-Aldrich, C9999) was used for a 10-min period, followed by natural cooling. The sections were then incubated in a blocking buffer (3% bovine serum albumin and 0.3% Triton X-100 in PBS) for 1 h at room temperature. Primary antibodies, including KI67 (Abcam, ab16667), GFP (Invitrogen, A11122), CD133 (Abcam, ab222782), CD56 (Abcam, ab237708), CD16 (Abcam, ab246222), IgM (Abcam, ab134159), and CD3 (Abcam, ab21703) were diluted in blocking solution and incubated overnight at 4 °C. After extensive washing with TBST (0.5% Tween 20 in TBS), the sections were incubated for 1 h with secondary antibodies conjugated to Alexa Fluor 488 and Alexa Fluor 555, followed by the addition of DAPI. Microscopic images were captured using the TissueGnostics panoramic tissue and cell quantitative analysis system. Immunofluorescence staining was analyzed using a confocal imaging system (Olympus FV3000 microscope).

### Western blot analysis

The tissues were homogenized using a grinder from Luca Sequencing Instrument Co., Ltd, Guangzhou, China. The homogenized tissues were then placed in a protein lysis buffer prepared by combining RIPA lysis buffer, protease inhibitor, and phosphatase inhibitor in a ratio of 98:1:1. After incubating the lysates on ice for 30 min, they were sonicated and centrifuged at maximum speed for 10 min. Total protein was extracted from the supernatant and quantified using the BCA protein quantification kit (Solarbio, Beijing, China). The proteins were subsequently separated by electrophoresis on 8% sodium dodecyl sulfate-polyacrylamide gels (SDS-PAGE) and transferred to polyvinylidene fluoride (PVDF) membranes. The PVDF membranes were blocked with 5% nonfat dry milk for 1 h and then incubated with primary antibodies overnight at 4 °C. Antibodies specific to RAG1 (Cell Signaling Technology, 3968 S), Vinculin (Sigma, MAB3574), and IL2RG (Invitrogen, PA5-80730) were used as primary antibodies. Finally, secondary antibodies labeled with horseradish peroxidase were applied, and the protein bands were detected using an electrochemiluminescence kit from CLINX. The results were analyzed using ImageJ software.

### Flow cytometry

T cells and B cells in the blood were examined using flow cytometry. EDTA-K3 tubes were used to collect blood samples for determining immunotypes and cell profiles. The BD FACS Verse flow cytometer system was employed for flow cytometry analysis to identify cell populations in the whole blood. A four-color immunofluorescence staining panel, consisting of CD4, CD3, IgM, and CD8 markers, was utilized in a single tube to detect different lymphocyte subsets. The antibodies used for detecting specific T cell subpopulations were employed at concentrations recommended by the manufacturer (refer to Supplementary Table 1 for antibody information). Data analysis was performed using FlowJo V10 software from FlowJo, Ashland, OR, USA.

### Monkey tumor formation experiment

MDA-MB-231-CMV-EGFP-Luc-Puro cells of human origin were cultivated in L-15 medium supplemented with 10% fetal bovine serum, 2 μg/mL puromycin, and 1% penicillin-streptomycin (all from Gibco) at a temperature of 37 °C in a humidified environment with 100% air. When the cells reached a confluence of 80–100%, the cell suspension was harvested and subjected to centrifugation at 300×*g* and 4 °C for 5 min. The cells were then resuspended in Matrigel at a concentration of 1 × 10^7^ cells/50 μL. Using an insulin syringe kept on ice, the mixture of cells and Matrigel was loaded. To conduct the procedure, the monkeys were anesthetized with isoflurane and placed in a supine position. The injection site in the axillary region was sterilized, and the cell suspension was slowly injected subcutaneously. Following the injection, the needle was held in place for 5 s. After complete recovery from anesthesia, the growth of the transplanted tumors was monitored. In accordance with approved protocols, the animals were euthanized, and the tumors were collected for in vitro assays.

### In vivo imaging systems

The study comprised both wild-type (WT) monkeys and monkeys subjected to base editing. To induce deep anesthesia, Zoletil^®^50 (4 mg/kg, Virbac, France) was administered to the animals through intramuscular injection. The AniView100 Multimode Live Animal Imaging System (Boluteng Biological Technology Co., Ltd, Guangzhou, China) was utilized to capture images of the monkeys. Following image acquisition, the data obtained were analyzed using the AniView software (Guangzhou, China), which is specifically designed for live monkey imaging.

### RNA-seq

To obtain total RNA, tissue samples underwent RNA extraction utilizing the Trizol reagent (Thermo Fisher Scientific, MA, USA). Sequencing libraries were generated using the SEQUMED® MustSeq® 3’mRNA DEG kit (Sequmed, China). The RNA-seq analysis was conducted at SequMed Bio Technology (Guangzhou, China) using the Illumina Novaseq 6000 platform (Illumina, San Diego, CA, USA). Data alignment and quantification were performed using the STAR aligner, enabling further analysis of the RNA-sequencing data.^44^

### RT-qPCR

Tissue samples were subjected to total RNA extraction using the Trizol method. The purity and integrity of the RNA were assessed, followed by reverse transcription using the PrimeScript™ RT Kit with gDNA Eraser (Takara, Kyoto, Japan). PCR amplification was subsequently performed using the TB Green Premix Ex Taq II (Takara, Kyoto, Japan). The resulting PCR products were detected using the CFX Connect Real-Time PCR Detection system (Bio-Rad, California, USA), with triplicate detection for each group. The relative expression of genes was determined using the cycle threshold (2^−^^ΔΔCt^) method.^45^ Please refer to Supplementary Table 2 for the primer sequences utilized in the RT-qPCR analysis.

### Potential off-target sites

To assess the possibility of off-target effects, we utilized Cas-OFFinder (http://www.rgenome.net/cas-offinder/)^46^ to predict and analyze potential off-target sites for each sgRNA. To validate the presence of off-target effects, PCR amplification of all predicted off-target sites was performed, followed by either Sanger sequencing or targeted deep sequencing. For a comprehensive list of the potential off-target sites and the corresponding primers used in the off-target assays, please refer to Supplementary Tables 3 and 4.

### Targeted deep sequence

To sequence the genomic loci of interest, we initiated by amplifying them from genomic DNA samples using amplification primers (Supplementary Tables 5 and 6) that contained Illumina forward and reverse adapters. The specific genomic region was amplified in the first round of PCR (PCR 1) using these primers. In a subsequent PCR reaction (PCR 2), unique Illumina barcoded primer pairs were incorporated into each sample. The PCR2 products, obtained by pooling the common amplicons, were purified using a gel extraction kit (Magen, Guangzhou) after 1.5% agarose gel electrophoresis and eluted with 40 μL of water. The concentration of DNA was measured through fluorescence quantification or qPCR, and sequencing was performed on an Illumina NovaSeq instrument following the manufacturer’s protocol.^43,47^ Annoroad Gene Technology Co., Ltd. (Beijing) conducted targeted deep sequencing, and the resulting data were analyzed using CRISPResso2 and custom R scripts.

### Statistics

Group comparisons were assessed for statistical significance using a two-tailed Student’s *t*-test. The data are presented as mean ± standard error of the mean (SEM). All calculations were conducted using GraphPad Prism 9 software. A significance level of *P* < 0.05 was deemed statistically significant.

### Supplementary information

Supplementary tables

Sigtrans_Supplementary_Materials_figures

## Data Availability

All the data supporting the results of the present study are provided to the corresponding authors upon request. The raw RNA-seq data are available from the NCBI Sequenced Read Archive (SRA) under accession code PRJNA993836. The raw data from deep sequencing are available from the NCBI Sequence Read Archive (SRA), with accession code PRJNA994260.
