# Supplementary material for: Generation of inactivated IL2RG and RAG1 monkeys with severe combined immunodeficiency using base editing
Source: Signal Transduct Target Ther. 2023 Sep 4;8:327. doi: 10.1038/s41392-023-01544-y (PMC10475462; doi:10.1038/s41392-023-01544-y)
Supplement: Supplementary file 1 — Supplementary tables [file 41392_2023_1544_MOESM1_ESM.docx]

Supplementary Materials for

**Engraftment of Tumor Cells with Inactivated IL2RG and RAG1 Monkeys Using Base Editing Accompanying Severe Combined Immunodeficiency**

Xiao Zheng^1,2^, Chunhui Huang^1,2^, Yingqi Lin^1,2^, Bofeng Han^1^, Yizi Chen^1^, Caijuan Li^1,2^, Jiawei Li^1,2^, Yongyan Ding^1^, Xichen Song^1^, Wei Wang^1^, Weien Liang^1^, Jianhao Wu^1^, Jiaxi Wu^1^, Jiale Gao^1^, Chengxi Wei^1^, Xudong Zhang^1^, Zhuchi Tu^1*^, Sen Yan^1,2*^

^1^Guangdong Key Laboratory of Non-human Primate Research, Guangdong-Hongkong-Macau Institute of CNS Regeneration, Jinan University, Guangzhou, 510632, China

^2^Department of Pathophysiology, School of Medicine, Jinan University, Guangzhou, 510632, China

Correspondence to: atuwater@163.com; 231yansen@163.com.

**This PDF file includes:**

Table. S1 to S6

**Supplementary table 1. Antibodies used in flow cytometry.**

| **Antibody** | **Label** | **Company** | **Catalog Number** |
| --- | --- | --- | --- |
| CD3 | APC | BD Biosciences | 557597 |
| CD4 | PE | BioLegend | 317410 |
| CD8 | PerCP-Cyanine5.5 | BioLegend | 301031 |
| IgM | FITC | BioLegend | 314506 |

**Supplementary table 2. All primer sequences used for RT-qPCR.**

| Primer Name | 5' to 3' |
| --- | --- |
| TP53-F | AGGGTTGGAAGTGTCTCATGC |
| TP53-R | CCTCTGACATTCTCGGAGCTT |
| CNN2-F | CAAAGGAAGCCTTCTCCCCTC |
| CNN2-R | CCCGGAGCGCAAATGTCCT |
| MKI67-F | TTACCAGGCGAAGGTATGAAAATG |
| MKI67-R | TGTTCCCTGAGCAACACTGTC |
| PPARG-F | AGCAAGAAGGCCATTTTCTCA |
| PPARG-R | GCAGGCTCCACTTTGATTGC |
| BCL2-F | TCATGTGTGTGGAGAGCGTC |
| BCL2-R | ACAGTTCCACAAAGGCGTCC |
| GATA3-F | CCCAGGTTTTACCTCTCGCT |
| GATA3-R | ACTCTGGAATTCTGCGAGCC |
| NME1-F | AGTGTAGTCGCCGGAGAGAG |
| NME1-R | CACACACGCCCGTCATTTAT |
| CDH2-F | CTTGGCTCTTCTCGCACGG |
| CDH2-R | ACTCTTCAAGCTGACTGGGTG |
| MMP2-F | TGATGGCATCGCTCAGATCC |
| MMP2-R | ACGACGGCATCCAGGTTATC |
| BRCA1-F | CTGAAAGCCAGGGAGTTGGT |
| BRCA1-R | TGTTAGAAGGCTGGCTTCCG |
| GAPDH-F | AGGTGAAGGTCGGAGTCAACGGAT |
| GAPDH-R | ATCTCGCTCCTGGAAGATGGTGAT |

**Supplementary table 3. The primers for targeted deep sequence of potential off-target sites used in this study at IL2RG and RAG1.**

| **Gene** | **Potential Off Target Site** | **Number of mismatch** | **Position** | **PCR Primer** | **Product length** | **potential off-target sites** |
| --- | --- | --- | --- | --- | --- | --- |
| IL2RG | GcTCAGCTCACAGGACCCACGAGG | 1 | chr9:+41819192 | F: TGGGCAGACCTGCACATTAC | 272 | OT1 |
|  |  |  |  | R: TTCCAAAGCAGGGATTCGCC |  |  |
|  | GTgTCAGCTCCAGGACCCACaGGG | 2 | chr6:+173766905 | F: CCTGGAACCGTATCAGGCATT | 271 | OT2 |
|  |  |  |  | R: ACCTGTCGGAGACTCGTGA |  |  |
|  | GcTCAGCTCCAGGACCCtCAGGGG | 2 | chr12:-105374363 | F: ACATGAGCCAGGGCTACTTTC | 250 | OT3 |
|  |  |  |  | R: GGCTCCACAGACCTTAACTGT |  |  |
|  | cTaCAGCTCCAGGCACCCAgGTGG | 3 | chr17:+94779186 | F: AGCCATTGCTTCACCTGTCC | 208 | OT4 |
|  |  |  |  | R: AATGCCGCCCATTCGTTAG |  |  |
|  | GgTaAGCTGCCAGGgCCCACGGGG | 3 | chr7:+155387664 | F: AATTCAGCCATAACAACCTAGCAAT | 269 | OT5 |
|  |  |  |  | R: TTAGCCGGTGAAATGCAGTG |  |  |
|  | GTGTCAGaTgaAGGACCCACGAGG | 3 | chr6:-149828436 | F: CGTCTCTGACCCCGAATAACTT | 203 | OT6 |
|  |  |  |  | R: CCGGCCGATTCGGTGC |  |  |
|  | GTTACAGaTCCAGGAtCCAgGGGG | 3 | chr6:+7613471 | F: GTGTGCAGTATTTTCTGAGAAGTCC | 230 | OT7 |
|  |  |  |  | R: TGTAAACCCGACTAATGGCCT |  |  |
|  | tTTCAGGCTCCAGGtCCCAgGAGG | 3 | chr15:+74000843 | F: TGAAAGTCGTGGCAAGACGG | 267 | OT8 |
|  |  |  |  | R: GCTAATGATGGGAAGAGAGGAAAAT |  |  |
|  | GTTgAGGgTCCgGGACCCACGCGG | 3 | chr3:+39915865 | F: CTGGTCAGAGGGGTGGTACT | 271 | OT9 |
|  |  |  |  | R: GTGTGCGGAGGACGTCTG |  |  |
| RAG1 | GcAGCAgTCTCCAGCAGGTCCTGGAGG | 2 | chr4:-34577678 | F: TGAAACAGAAGGGAGGCGAC | 236 | OT1 |
|  |  |  |  | R: AGGGCCCCCTGAAATCCTAT |  |  |
|  | GGAGCAATCgCtAGGaAGTCCTGGTGG | 3 | chr20:+2521136 | F: ATCTTTGAGAGTTCGTCGCC | 206 | OT2 |
|  |  |  |  | R: TGCCTCCCCATCTACAAATCT |  |  |
|  | GcAGCAGgTCTCCAGgAGTCCTGGAGG | 3 | chr11:-121552326 | F: CCCAGGGATCTGCTATTCTAGG | 223 | OT3 |
|  |  |  |  | R: TGACCACGGGACTAGGGTCTA |  |  |
|  | GcAGCAgTCTCCAGCAGTCC--cAGG | 3 | chr7:+115276107 | F: GGAATCGGTCTGAGAGAGTTGG | 204 | OT4 |
|  |  |  |  | R: TGTCCTCTAGGAGGAAAATCACCT |  |  |
|  | GGgGCcgTCTCCAG-AGTCCTGGAGG | 3 | chr15:+4186788 | F: CTTGTTCTCAGAAAGGCGGC | 271 | OT5 |
|  |  |  |  | R: TGCGAACTCGTTATCGGAGG |  |  |
|  | GagaCAATCTCCAGCA-TCCTGGAGG | 3 | chr15:-73824741 | F: ACACTGCCAAGTAGGTGAGC | 256 | OT6 |
|  |  |  |  | R: ACCAGCAGCCAAAGAGATCC |  |  |
|  | GaAcCAATCTCCtGCAAAGTCCTGGGGG | 3 | chr3:-81672526 | F: AGAAGCCCTAACAACTTCCCTG | 277 | OT7 |
|  |  |  |  | R: TGCCCAGTATTCTGGTTGGC |  |  |
|  | GGAGgcATCTCCAGCAGTCCT-GCGG | 3 | chr8:+75980512 | F: GCCTGGGCCATCTCTCTTAC | 214 | OT8 |
|  |  |  |  | R: CTCAACCCTTAGGCCCTTGG |  |  |
|  | GGAGCAtTCTgCAGCAG-CCTaGAGG | 3 | chr9:-36789618 | F: AGCCCCCTAAGGAAGGAACT | 236 | OT9 |
|  |  |  |  | R: GCACAGCCTCTCCACAGATT |  |  |

**Supplementary table 4. The primers for Sanger sequencing of potential off-target sites used in this study at IL2RG and RAG1.**

| **Gene** | **Potential Off Target Site** | **Number of mismatch** | **Position** | **PCR Primer** | **Product length** | **potential off-target sites** |
| --- | --- | --- | --- | --- | --- | --- |
| IL2RG | GcTCAGCTCACAGGACCCACGAGG | 1 | chr9:+41819192 | F: GGGAATGTGTGCTTGTCCAC | 272 | OT1 |
|  |  |  |  | R: GTGCTGCCATTCCATGACTC |  |  |
|  | GgTCAGCTCCATGGAaCCACGTGG | 2 | chr13:+100239155 | F: GAATCCGGCGAAATGCCAC | 280 | OT10 |
|  |  |  |  | R: GCTTGGTGATCATCGCTTGC |  |  |
|  | GTgTCAGCTCCAGGACCCACaGGG | 2 | chr6:+173766905 | F: GTCTTTTGCCCACTTCGTTGT | 271 | OT2 |
|  |  |  |  | R: TGACCTGTCGGAGACTCGTG |  |  |
|  | GcTCAGCTCCAGGACCCtCAGGGG | 2 | chr12:-105374363 | F: AGGGACTTGCCCTGATTCAC | 250 | OT3 |
|  |  |  |  | R: GAAGTAGGGAGGCTCCACAG |  |  |
|  | GTTCcaCTCCAGGAGCCCACGTGG | 2 | chr20:+38459594 | F: ACCATCCAGCATCTGCATTTTAGT | 297 | OT11 |
|  |  |  |  | R: AGTACCTGCGATTCCACTGAAG |  |  |
|  | cTaCAGCTCCAGGCACCCAgGTGG | 3 | chr17:+94779186 | F: AGCCATTGCTTCACCTGTCC | 208 | OT4 |
|  |  |  |  | R: AATGCCGCCCATTCGTTAG |  |  |
|  | GTCTCAGCTCCAGGgCatACGAGG | 3 | chr7:+125375582 | F: GATCCCGATGTGGAGAATGCTG | 223 | OT12 |
|  |  |  |  | R: AGAAAAAGTTCCTCGTATGCCCT |  |  |
|  | GTTCgGCTCCAGGcCCCATCaAGG | 3 | chr7:-99403784 | F: TTTTCGTCACTCTTCCCAGATG | 204 | OT13 |
|  |  |  |  | R: CCAGACAAGGAGTGTTCGGC |  |  |
|  | GcTCAGCTCCAGGgCCCAtCGCGG | 3 | chr13:+109108972 | F: CTGCGTCCCTCAAAGGGAAT | 239 | OT14 |
|  |  |  |  | R: TGCTGTGTAGACGCTCATGG |  |  |
|  | tgTCAGCTCCAGGACCCACaGGG | 3 | chr6:+173766906 | F: GTCTTTTGCCCACTTCGTTGT | 281 | OT15 |
|  |  |  |  | R: ACCCTTTACATGACCTGTCGG |  |  |
|  | GTTACAGaTCCAGGAtCCAgGGGG | 3 | chr6:+7613471 | F: GTGTGCAGTATTTTCTGAGAAGTCC | 230 | OT7 |
|  |  |  |  | R: TGTAAACCCGACTAATGGCCT |  |  |
|  | GTTgAGGgTCCgGGACCCACGCGG | 3 | chr3:+39915865 | F: CTGGTCAGAGGGGTGGTACT | 271 | OT9 |
|  |  |  |  | R: GTGTGCGGAGGACGTCTG |  |  |
| RAG1 | GcAGCAgTCTCCAGCAGGTCCTGGAGG | 2 | chr4:-34577678 | F: TGAAACAGAAGGGAGGCGAC | 236 | OT1 |
|  |  |  |  | R: AGGGCCCCCTGAAATCCTAT |  |  |
|  | GaAcCAATCTCCtGCAAAGTCCTGGGGG | 3 | chr3:-81672526 | F: CCTAACAACTTCCCTGAATCTGCT | 299 | OT7 |
|  |  |  |  | R: TGTCCTAGTTTCTCTGCATCAGG |  |  |
|  | GGAGCAATCgCtAGGaAGTCCTGGTGG | 3 | chr20:+2521136 | F: ATCTTTGAGAGTTCGTCGCC | 206 | OT2 |
|  |  |  |  | R: TGCCTCCCCATCTACAAATCT |  |  |
|  | GGAGCccTCTCCAGgAGCTCCTGGTGG | 3 | chr20:+71835018 | F: GGCTAGTGTGCTGGTATCCG | 251 | OT10 |
|  |  |  |  | R: CTGGCAGCAGATCTTAACACG |  |  |
|  | GcAGCAGgTCTCCAGgAGTCCTGGAGG | 3 | chr11:-121552326 | F: CCCAGGGATCTGCTATTCTAGG | 223 | OT3 |
|  |  |  |  | R: TGACCACGGGACTAGGGTCTA |  |  |
|  | GcAGCAgTCTCCAGCAGTCC--cAGG | 3 | chr7:+115276107 | F: GGAATCGGTCTGAGAGAGTTGG | 204 | OT4 |
|  |  |  |  | R: TGTCCTCTAGGAGGAAAATCACCT |  |  |
|  | GGgGCcgTCTCCAG-AGTCCTGGAGG | 3 | chr15:+4186788 | F: CTTGTTCTCAGAAAGGCGGC | 271 | OT5 |
|  |  |  |  | R: TGCGAACTCGTTATCGGAGG |  |  |
|  | GagaCAATCTCCAGCA-TCCTGGAGG | 3 | chr15:-73824741 | F: ACACTGCCAAGTAGGTGAGC | 256 | OT6 |
|  |  |  |  | R: ACCAGCAGCCAAAGAGATCC |  |  |
|  | GaAcCAATCTCCtGCAAAGTCCTGGGGG | 3 | chr3:-81672526 | F: AGAAGCCCTAACAACTTCCCTG | 277 | OT12 |
|  |  |  |  | R: TGCCCAGTATTCTGGTTGGC |  |  |
|  | GGAGgcATCTCCAGCAGTCCT-GCGG | 3 | chr8:+75980512 | F: GCCTGGGCCATCTCTCTTAC | 214 | OT8 |
|  |  |  |  | R: CTCAACCCTTAGGCCCTTGG |  |  |
|  | GGAGgAATCTaCAGgAGTC-TGGAGG | 3 | chr1:-188972139 | F: AAATGTGTGGTGGATGCGAC | 200 | OT11 |
|  |  |  |  | R: GGACTCTACAGCGAATGGAGG |  |  |
|  | GGAGCAtTCTgCAGCAG-CCTaGAGG | 3 | chr9:-36789618 | F: AGCCCCCTAAGGAAGGAACT | 236 | OT9 |
|  |  |  |  | R: GCACAGCCTCTCCACAGATT |  |  |
|  | GGAaCAATCTCCCAGCtGTCaTGGTGG | 3 | chrX:-7836421 | F: CCTGCAGTTTTGCGATGCTT | 296 | OT13 |
|  |  |  |  | R: CACTCTGACCAACACAGGGG |  |  |

**Supplementary table 5. Targeted deep sequence of IL2RG**

| **Name** | **Off-target sites** | **PCR product sequencing results** | **The first round of PCR primers**  **5’-3’** | **Second round of PCR primers**  **5’-3’** |
| --- | --- | --- | --- | --- |
| OT1 | GcTCAGCTCACAGGACCCACGAGG | AATGATACGGCGACCACCGAGATCTACACTAGATCGCTCGTCGGCAGCGTCAGATGTGTATAAGAGACAGtgggcagacctgcacattacggcctcgtgggtcctgtgagctgagcctcagtgttcccatctgagaaacgggcctctctgggctgcctccctggatgagggagaattgcattgagatgagtcatggaatggcagcacacagctcactctaactcctaatgatgcacacttaaagcaaagcaaagttagtcaagtgaaggcgaatccctgctttggaaCTGTCTCTTATACACATCTCCGAGCCCACGAGACGTGTGTCGATCTCGTATGCCGTCTTCTGCTTG | Forword: TCGTCGGCAGCGTCAGATGTGTATAAGAGACAGTGGGCAGACCTGCACATTAC  Reverse: GTCTCGTGGGCTCGGAGATGTGTATAAGAGACAGTTCCAAAGCAGGGATTCGCC | P5: AATGATACGGCGACCACCGAGATCTACACTAGATCGCTCGTCGGCAGCGTC  P7:  CAAGCAGAAGACGGCATACGAGATCGACACACGTCTCGTGGGCTCGG |
| OT2 | GTgTCAGCTCCAGGACCCACaGGG | AATGATACGGCGACCACCGAGATCTACACTAGATCGCTCGTCGGCAGCGTCAGATGTGTATAAGAGACAGcctggaaccgtatcaggcattcccctgcctctcataaacatttcagggaggccagttctgcagggtgtcagctccaggacccacagggccagaaccggctgggagagttggttatttgagatgtggtaccgcttcctcacgagtctccgacaggtCTGTCTCTTATACACATCTCCGAGCCCACGAGACGTACATCTATCTCGTATGCCGTCTTCTGCTTG | Forword: TCGTCGGCAGCGTCAGATGTGTATAAGAGACAGCCTGGAACCGTATCAGGCATT  Reverse: GTCTCGTGGGCTCGGAGATGTGTATAAGAGACAGACCTGTCGGAGACTCGTGA | P5: AATGATACGGCGACCACCGAGATCTACACTAGATCGCTCGTCGGCAGCGTC  P7:  CAAGCAGAAGACGGCATACGAGAT AGATGTACGTCTCGTGGGCTCGG |
| OT3 | GcTCAGCTCCAGGACCCtCAGGGG | AATGATACGGCGACCACCGAGATCTACACTAGATCGCTCGTCGGCAGCGTCAGATGTGTATAAGAGACAGacatgagccagggctactttcttcagggctgaatgcctaaatgaagctgggctaatcatattgtctctcctggaaatgtggacatctgcgttcagaggaccggcccctgagggtcctggagctgagccacagagcatcctaaagggaaagtccagatattcccacagttaaggtctgtggagccCTGTCTCTTATACACATCTCCGAGCCCACGAGACGAATCTGTATCTCGTATGCCGTCTTCTGCTTG | Forword: TCGTCGGCAGCGTCAGATGTGTATAAGAGACAGACATGAGCCAGGGCTACTTTC  Reverse: GTCTCGTGGGCTCGGAGATGTGTATAAGAGACAGGGCTCCACAGACCTTAACTGT | P5: AATGATACGGCGACCACCGAGATCTACACTAGATCGCTCGTCGGCAGCGTC  P7:  CAAGCAGAAGACGGCATACGAGAT ACAGATTCGTCTCGTGGGCTCGG |
| OT4 | cTaCAGCTCCAGGCACCCAgGTGG | AATGATACGGCGACCACCGAGATCTACACTAGATCGCTCGTCGGCAGCGTCAGATGTGTATAAGAGACAGagccattgcttcacctgtcctgtccatcaacccctctgccagtatcatacggtaaatagtaccatgaatactacagctccaggcacccaggtggcatcagagaactgacaaattagagtttaacgatatcctcagtttgtagccggggctgcttcagagctgatgactggggggtattgctgtctggagctaacgaatgggcggcattCTGTCTCTTATACACATCTCCGAGCCCACGAGACGATTCATCATCTCGTATGCCGTCTTCTGCTTG | Forword: TCGTCGGCAGCGTCAGATGTGTATAAGAGACAGAGCCATTGCTTCACCTGTCC  Reverse: GTCTCGTGGGCTCGGAGATGTGTATAAGAGACAGAATGCCGCCCATTCGTTAG | P5: AATGATACGGCGACCACCGAGATCTACACTAGATCGCTCGTCGGCAGCGTC  P7:  CAAGCAGAAGACGGCATACGAGAT GATGAATCGTCTCGTGGGCTCGG |
| OT5 | GgTaAGCTGCCAGGgCCCACGGGG | AATGATACGGCGACCACCGAGATCTACACTAGATCGCTCGTCGGCAGCGTCAGATGTGTATAAGAGACAGaattcagccataacaacctagcaataacaataccactgctactatcatcctcattgacaggcaaggaaactgaggcacagagagggtaagctgccagggcccacggggaggaagaatcagacctgatgcagacctggacgtgtggctctgagcctgtgcatcactcacagtgccctgccgccaggaagtgaggggagctcatggggagcccacgggctggatgaggaggtcagaccaaggaagagggaacactgcatttcaccggctaaCTGTCTCTTATACACATCTCCGAGCCCACGAGACGCTAACTCATCTCGTATGCCGTCTTCTGCTTG | Forword: TCGTCGGCAGCGTCAGATGTGTATAAGAGACAGAATTCAGCCATAACAACCTAGCAAT  Reverse: GTCTCGTGGGCTCGGAGATGTGTATAAGAGACAGTTAGCCGGTGAAATGCAGTG | P5: AATGATACGGCGACCACCGAGATCTACACTAGATCGCTCGTCGGCAGCGTC  P7:  CAAGCAGAAGACGGCATACGAGAT GAGTTAGCGTCTCGTGGGCTCGG |
| OT6 | GTGTCAGaTgaAGGACCCACGAGG  反向 | AATGATACGGCGACCACCGAGATCTACACTAGATCGCTCGTCGGCAGCGTCAGATGTGTATAAGAGACAGcgtctctgaccccgaataacttgttggtttccccccgggctttgtttcccggggtcaccacctcggggacgccgggcagccgccccggccctcgtgggtccttcatctgacaccccctccggcccccgccccctacccgctcggagataggggtcaaaggcagcccgcgtcacgtggtcgccgagcagcaccgaatcggccggCTGTCTCTTATACACATCTCCGAGCCCACGAGACGAGGTGCTATCTCGTATGCCGTCTTCTGCTTG | Forword: TCGTCGGCAGCGTCAGATGTGTATAAGAGACAGCGTCTCTGACCCCGAATAACTT  Reverse: GTCTCGTGGGCTCGGAGATGTGTATAAGAGACAGCCGGCCGATTCGGTGC | P5: AATGATACGGCGACCACCGAGATCTACACTAGATCGCTCGTCGGCAGCGTC  P7:  CAAGCAGAAGACGGCATACGAGAT AGCACCTCGTCTCGTGGGCTCGG |
| OT7 | GTTACAGaTCCAGGAtCCAgGGGG | AATGATACGGCGACCACCGAGATCTACACTAGATCGCTCGTCGGCAGCGTCAGATGTGTATAAGAGACAGgtgtgcagtattttctgagaagtcctttagttagaagggttgagatcaccacatatttgcaaagattgcagtgaaggttttgctatgactgtgcactcaacctcctttaaggagaaatcaatagttacagatccaggatccagggggaggatgaaggaactcgcaggaatcactatccagtcttgatgcctaacaaagaacataaatgcaggccattagtcgggtttacaCTGTCTCTTATACACATCTCCGAGCCCACGAGACGCACTGTCATCTCGTATGCCGTCTTCTGCTTG | Forword: TCGTCGGCAGCGTCAGATGTGTATAAGAGACAGGTGTGCAGTATTTTCTGAGAAGTCC  Reverse: GTCTCGTGGGCTCGGAGATGTGTATAAGAGACAGTGTAAACCCGACTAATGGCCT | P5: AATGATACGGCGACCACCGAGATCTACACTAGATCGCTCGTCGGCAGCGTC  P7:  CAAGCAGAAGACGGCATACGAGAT GACAGTGCGTCTCGTGGGCTCGG |
| OT8 | TTTCAGGCTCCAGGtCCCAgGAGG | AATGATACGGCGACCACCGAGATCTACACTAGATCGCTCGTCGGCAGCGTCAGATGTGTATAAGAGACAGtgaaagtcgtggcaagacggcggctccacctctggcctgtgatcctcctcctgggacctggagcctgaaacctcttatgttctttatatcctttaccttctcacttcactcccagctgttttgaaatcctctttattcctttttcctccatggtgcagatctgggattcagaaatcaggatcttagtgtgactactttgaaattatcagcatcactcttaatcttctctgaatatgatttttattttcctctcttcccatcattagcCTGTCTCTTATACACATCTCCGAGCCCACGAGACGCCTGTTCATCTCGTATGCCGTCTTCTGCTTG | Forword: TCGTCGGCAGCGTCAGATGTGTATAAGAGACAGTGAAAGTCGTGGCAAGACGG  Reverse: GTCTCGTGGGCTCGGAGATGTGTATAAGAGACAGGCTAATGATGGGAAGAGAGGAAAAT | P5: AATGATACGGCGACCACCGAGATCTACACTAGATCGCTCGTCGGCAGCGTC  P7:  CAAGCAGAAGACGGCATACGAGAT GAACAGGCGTCTCGTGGGCTCGG |
| OT9 | GTTgAGGgTCCgGGACCCACGCGG | AATGATACGGCGACCACCGAGATCTACACTAGATCGCTCGTCGGCAGCGTCAGATGTGTATAAGAGACAGtggtcagaggggtggtactcggaggccggcgagcgcgggcaagggcggcccagcctgcggggatccggtgtccgcgctgccgccccccgcgagcgcctgagcctggctgtccgcgtgggtcccggaccctcaactccaggagactctcgtccctcagattccccggctgtgccctggagggggacgttcgtccggggagcgctgcaggggagctgggcgtagggttccgcgcgctctcccacctgggcgggccagacgtcctccgcacacCTGTCTCTTATACACATCTCCGAGCCCACGAGACGCAATCCGATCTCGTATGCCGTCTTCTGCTTG | Forword: TCGTCGGCAGCGTCAGATGTGTATAAGAGACAGCTGGTCAGAGGGGTGGTACT  Reverse: GTCTCGTGGGCTCGGAGATGTGTATAAGAGACAGGTGTGCGGAGGACGTCTG | P5: AATGATACGGCGACCACCGAGATCTACACTAGATCGCTCGTCGGCAGCGTC  P7:  CAAGCAGAAGACGGCATACGAGAT CGGATTGCGTCTCGTGGGCTCGG |

**Supplementary table 6. Targeted deep sequence of RAG1**

| **Name** | **Off-target sites** | **PCR product sequencing results** | **The first round of PCR primers**  **5’-3’** | **Second round of PCR primers**  **5’-3’** |
| --- | --- | --- | --- | --- |
| OT1 | GcAGCAgTCTCCAGCAGGTCCTGGAGG | AATGATACGGCGACCACCGAGATCTACACTAGATCGCTCGTCGGCAGCGTCAGATGTGTATAAGAGACAGtgaaacagaagggaggcgactttctaaaacaagattctttttggagggtggaaggaggtggggaaaggaatgccgttactagagaaacatgacttgaggcagcagtctccagcaggtcctggaggatcagggagactccttggtgcttgcccagtggtcacagcctggccatgaaggatggaagaagaaatgagtaggaagggaaagacaataagcataggatttcagggggccctCTGTCTCTTATACACATCTCCGAGCCCACGAGACGGATTAGGATCTCGTATGCCGTCTTCTGCTTG | Forword: TCGTCGGCAGCGTCAGATGTGTATAAGAGACAGTGAAACAGAAGGGAGGCGAC  Reverse: GTCTCGTGGGCTCGGAGATGTGTATAAGAGACAGAGGGCCCCCTGAAATCCTAT | P5: AATGATACGGCGACCACCGAGATCTACACTAGATCGCTCGTCGGCAGCGTC  P7:  CAAGCAGAAGACGGCATACGAGAT CCTAATCCGTCTCGTGGGCTCGG |
| OT2 | GGAGCAATCgCtAGGaAGTCCTGGTGG | AATGATACGGCGACCACCGAGATCTACACTAGATCGCTCGTCGGCAGCGTCAGATGTGTATAAGAGACAGatctttgagagttcgtcgccctggaatacgtttggcttttgcagctcttttaattgctgacaggaagacccgggtcgggagaggggagcaaggagcaatcgctaggaagtcctggtggggagggatttgggcccacagaggggagtggcgtttcctttcaggactggaagaatggatagaaaggtagatttgtagatggggaggcaCTGTCTCTTATACACATCTCCGAGCCCACGAGACGAGGATGGATCTCGTATGCCGTCTTCTGCTTG | Forword: TCGTCGGCAGCGTCAGATGTGTATAAGAGACAGATCTTTGAGAGTTCGTCGCC  Reverse: GTCTCGTGGGCTCGGAGATGTGTATAAGAGACAGTGCCTCCCCATCTACAAATCT | P5: AATGATACGGCGACCACCGAGATCTACACTAGATCGCTCGTCGGCAGCGTC  P7:  CAAGCAGAAGACGGCATACGAGAT CCATCCTCGTCTCGTGGGCTCGG |
| OT3 | GcAGCAGgTCTCCAGgAGTCCTGGAGG | AATGATACGGCGACCACCGAGATCTACACTAGATCGCTCGTCGGCAGCGTCAGATGTGTATAAGAGACAGcccagggatctgctattctaggcagaaggagatgctgagaactaaggcccagtgtgactgatcctcttgccactggcctcccagagagggaggcggtctcctccaggactcctggagacctgctgcagctgagggcttgtggcaatcaaacttggtaatgggtgtagtagaagatctggtcacactcagctgcttcaaggcttagaccctagtcccgtggtcaCTGTCTCTTATACACATCTCCGAGCCCACGAGACGTAAGGTGATCTCGTATGCCGTCTTCTGCTTG | Forword: TCGTCGGCAGCGTCAGATGTGTATAAGAGACAGCCCAGGGATCTGCTATTCTAGG  Reverse: GTCTCGTGGGCTCGGAGATGTGTATAAGAGACAGTGACCACGGGACTAGGGTCTA | P5: AATGATACGGCGACCACCGAGATCTACACTAGATCGCTCGTCGGCAGCGTC  P7:  CAAGCAGAAGACGGCATACGAGAT CACCTTACGTCTCGTGGGCTCGG |
| OT4 | GcAGCAgTCTCCAGCAGTCC--cAGG | AATGATACGGCGACCACCGAGATCTACACTAGATCGCTCGTCGGCAGCGTCAGATGTGTATAAGAGACAGggaatcggtctgagagagttgggctttcttccaagtgcgtgaatcgacctttcttagatcaattccatttccagagcccatgcagcagtctccagcagtcccaggaagagagaaagatgagtcataaagtgcccttaagtggttcatctgtgttaatctagaccactggttctcaactggaggtgattttcctcctagaggacaCTGTCTCTTATACACATCTCCGAGCCCACGAGACGCTCCTTGATCTCGTATGCCGTCTTCTGCTTG | Forword: TCGTCGGCAGCGTCAGATGTGTATAAGAGACAGGGAATCGGTCTGAGAGAGTTGG  Reverse: GTCTCGTGGGCTCGGAGATGTGTATAAGAGACAGTGTCCTCTAGGAGGAAAATCACCT | P5: AATGATACGGCGACCACCGAGATCTACACTAGATCGCTCGTCGGCAGCGTC  P7:  CAAGCAGAAGACGGCATACGAGAT CAAGGAGCGTCTCGTGGGCTCGG |
| OT5 | GGgGCcgTCTCCAG-AGTCCTGGAGG | AATGATACGGCGACCACCGAGATCTACACTAGATCGCTCGTCGGCAGCGTCAGATGTGTATAAGAGACAGcttgttctcagaaaggcggccaggcagacctgagggagggcagaggggtcagcagagagggcagcacgggggccgtctccagagtcctggagggaggatctccgggctctggggaaccctggagactcccaagtaccaacagaggcctgctcgcttccccagcctcacccgtagggctgggcagagtgcaggggtctggccccagggcaaggctggggtgccggctgtgaggtgaggggcaccagctgccccctccgataacgagttcgcaCTGTCTCTTATACACATCTCCGAGCCCACGAGACGAGCCAATATCTCGTATGCCGTCTTCTGCTTG | Forword: TCGTCGGCAGCGTCAGATGTGTATAAGAGACAGCTTGTTCTCAGAAAGGCGGC  Reverse: GTCTCGTGGGCTCGGAGATGTGTATAAGAGACAGTGCGAACTCGTTATCGGAGG | P5: AATGATACGGCGACCACCGAGATCTACACTAGATCGCTCGTCGGCAGCGTC  P7:  CAAGCAGAAGACGGCATACGAGAT ATTGGCTCGTCTCGTGGGCTCGG |
| OT6 | GagaCAATCTCCAGCA-TCCTGGAGG | AATGATACGGCGACCACCGAGATCTACACTAGATCGCTCGTCGGCAGCGTCAGATGTGTATAAGAGACAGacactgccaagtaggtgagcaatgaaaaggtcaagttatatggcatcgaatctaaagagatcgttatgatcacgtacaaaacccccaggagacaatctccagcatcctggaggagcagttgctggaatctgaaccactcacaggtaagaactccagcccctgccgccttcctgcacttcccattgtttggccccgggctgctgaccaattggaatgcagtctgcaagtcaggattcggatctctttggctgctggtCTGTCTCTTATACACATCTCCGAGCCCACGAGACGGAATGATATCTCGTATGCCGTCTTCTGCTTG | Forword: TCGTCGGCAGCGTCAGATGTGTATAAGAGACAGACACTGCCAAGTAGGTGAGC  Reverse: GTCTCGTGGGCTCGGAGATGTGTATAAGAGACAGACCAGCAGCCAAAGAGATCC | P5: AATGATACGGCGACCACCGAGATCTACACTAGATCGCTCGTCGGCAGCGTC  P7:  CAAGCAGAAGACGGCATACGAGAT ATCATTCCGTCTCGTGGGCTCGG |
| OT7 | GaAcCAATCTCCtGCAAAGTCCTGGGGG | AATGATACGGCGACCACCGAGATCTACACTAGATCGCTCGTCGGCAGCGTCAGATGTGTATAAGAGACAGagaagccctaacaacttccctgaatctgctaacaaaaatacctaatgaagaaaaagagaaaattcctaacaattggccatagaaccaatctcctgcaaagtcctgggggaacttctcattagctacaaaccagagagccatgtaccttagaagtacgtagtatgtgttttttattacctgaaatagaggaaacctaccctcatgaaacagtaagggatgtaatcattctccactttagcatgaaactctatgtatttgccaaccagaatactgggcaCTGTCTCTTATACACATCTCCGAGCCCACGAGACGTCGCTATATCTCGTATGCCGTCTTCTGCTTG | Forword: TCGTCGGCAGCGTCAGATGTGTATAAGAGACAGAGAAGCCCTAACAACTTCCCTG  Reverse: GTCTCGTGGGCTCGGAGATGTGTATAAGAGACAGTGCCCAGTATTCTGGTTGGC | P5: AATGATACGGCGACCACCGAGATCTACACTAGATCGCTCGTCGGCAGCGTC  P7:  CAAGCAGAAGACGGCATACGAGAT ATAGCGACGTCTCGTGGGCTCGG |
| OT8 | GGAGgcATCTCCAGCAGTCCT-GCGG | AATGATACGGCGACCACCGAGATCTACACTAGATCGCTCGTCGGCAGCGTCAGATGTGTATAAGAGACAGgcctgggccatctctcttactgtggcctggggctggcacttggttgttgtgctgttcatacatgaatgtggcttcactccgcaggactgctggagatgcctccaaagcagtgagcagctctgcacctctggccaagcccctggctagagacccccaacccatccctgctggttgctactgcccctccccaaatcccaagggcctaagggttgagCTGTCTCTTATACACATCTCCGAGCCCACGAGACGTTAGCCTATCTCGTATGCCGTCTTCTGCTTG | Forword: TCGTCGGCAGCGTCAGATGTGTATAAGAGACAGGCCTGGGCCATCTCTCTTAC  Reverse: GTCTCGTGGGCTCGGAGATGTGTATAAGAGACAGCTCAACCCTTAGGCCCTTGG | P5: AATGATACGGCGACCACCGAGATCTACACTAGATCGCTCGTCGGCAGCGTC  P7:  CAAGCAGAAGACGGCATACGAGAT AGGCTAACGTCTCGTGGGCTCGG |
| OT9 | GGAGCAtTCTgCAGCAG-CCTaGAGG | AATGATACGGCGACCACCGAGATCTACACTAGATCGCTCGTCGGCAGCGTCAGATGTGTATAAGAGACAGagccccctaaggaaggaacttgttgattttggcagtgtttacctttcctacaaacaaattcgacattttccagactggctggctcttaaaaatcacttgagggctggggaaaagctgcctctaggctgctgcagaatgctccctgctctgttctaggaaaatctttgtgcccttcacagctgctggtggataatctatggagtcatgattatttagaatctgtggagaggctgtgcCTGTCTCTTATACACATCTCCGAGCCCACGAGACGCATGGCTATCTCGTATGCCGTCTTCTGCTTG | Forword: TCGTCGGCAGCGTCAGATGTGTATAAGAGACAGAGCCCCCTAAGGAAGGAACT  Reverse: GTCTCGTGGGCTCGGAGATGTGTATAAGAGACAGGCACAGCCTCTCCACAGATT | P5: AATGATACGGCGACCACCGAGATCTACACTAGATCGCTCGTCGGCAGCGTC  P7:  CAAGCAGAAGACGGCATACGAGAT AGCCATGCGTCTCGTGGGCTCGG |
