# Supplementary material for: Generation of inactivated IL2RG and RAG1 monkeys with severe combined immunodeficiency using base editing
Source: Signal Transduct Target Ther. 2023 Sep 4;8:327. doi: 10.1038/s41392-023-01544-y (PMC10475462; doi:10.1038/s41392-023-01544-y)
Supplement: Supplementary file 2 — Sigtrans_Supplementary_Materials_figures [file 41392_2023_1544_MOESM2_ESM.docx]

Supplementary Materials for

**Engraftment of Tumor Cells with Inactivated IL2RG and RAG1 Monkeys Using Base Editing Accompanying Severe Combined Immunodeficiency**

Xiao Zheng^1,2^, Chunhui Huang^1,2^, Yingqi Lin^1,2^, Bofeng Han^1^, Yizhi Chen^1^, Caijuan Li^1,2^, Jiawei Li^1,2^, Yongyan Ding^1^, Xichen Song^1^, Wei Wang^1^, Weien Liang^1^, Jianhao Wu^1^, Jiaxi Wu^1^, Jiale Gao^1^, Chengxi Wei^1^, Xudong Zhang^1^, Zhuchi Tu^1*^, Sen Yan^1,2*^

^1^Guangdong Key Laboratory of Non-human Primate Research, Guangdong-Hongkong-Macau Institute of CNS Regeneration, Jinan University, Guangzhou, 510632, China

^2^Department of Pathophysiology, School of Medicine, Jinan University, Guangzhou, 510632, China

Correspondence to: atuwater@163.com; 231yansen@163.com.

**This PDF file includes:**

Figures. S1 to S6


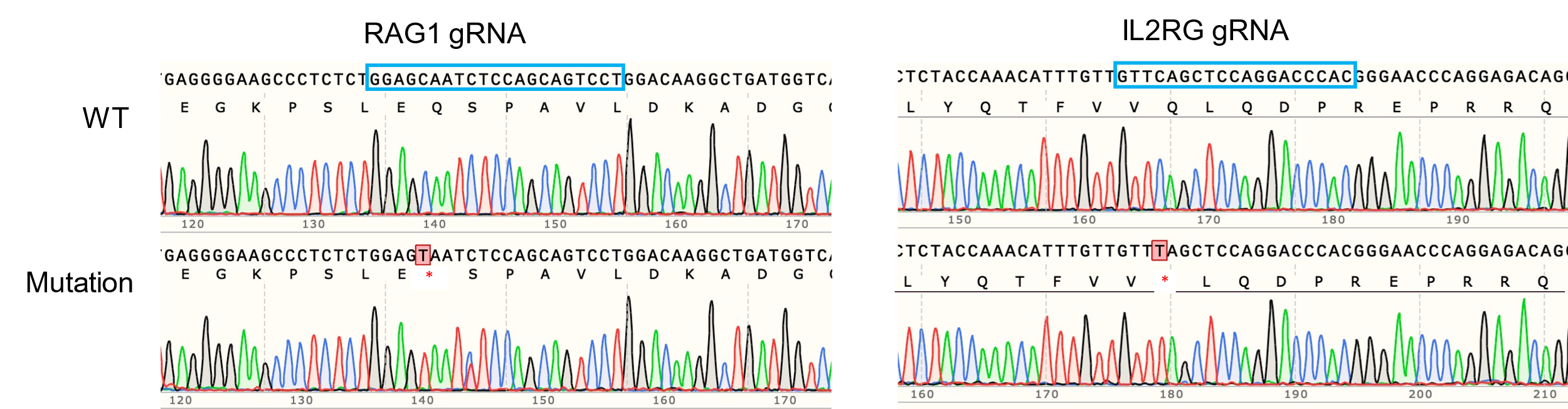
**Supplementary Figure captions and legends**

**Fig. S1 Genotypes of base-edited embryos.** Red stars indicate mutated base.


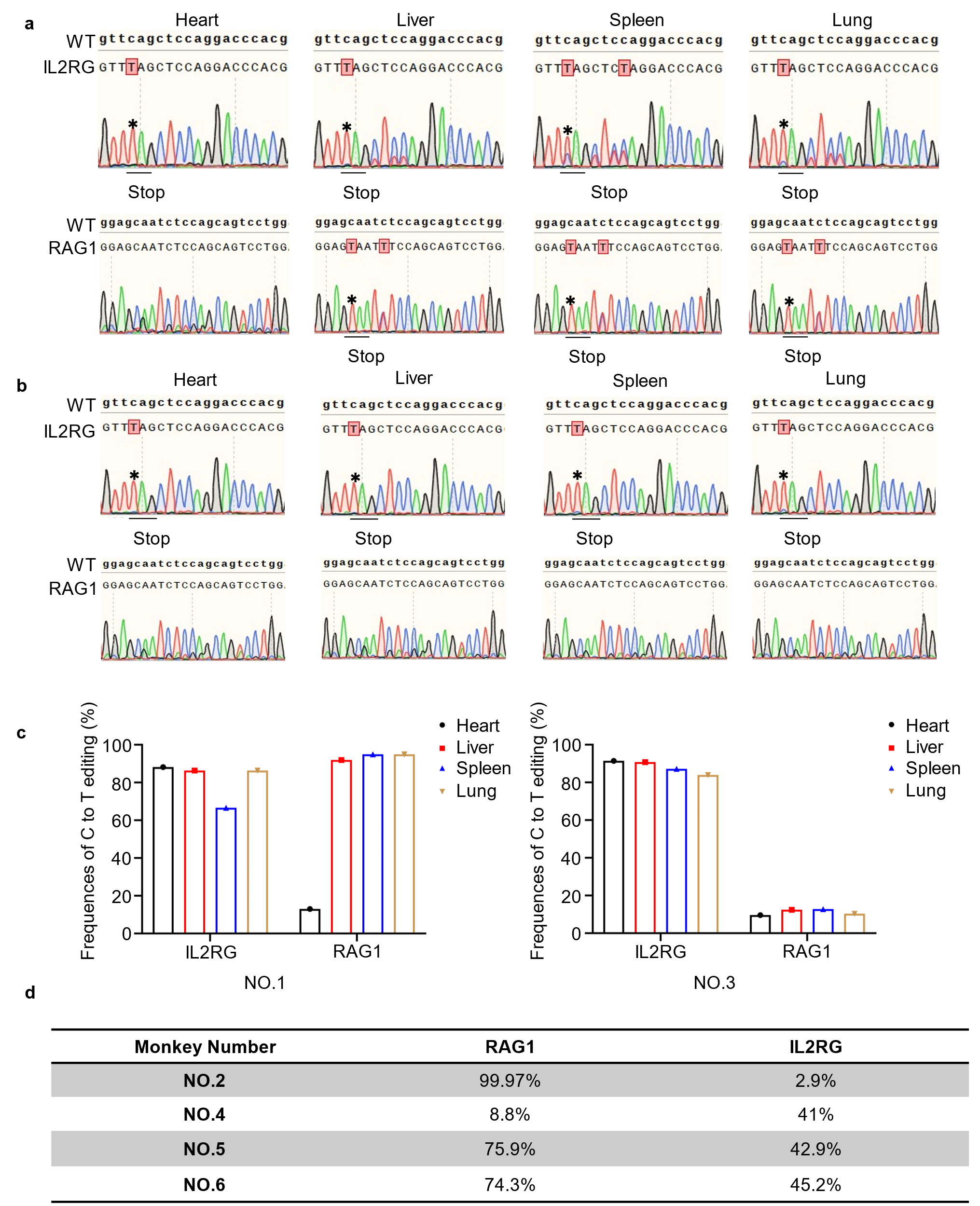


**Fig. S2 Editing efficiency in monkeys generated using the CBE4max system.**

**a-b** Sanger sequencing for validation of editing efficiency in heart, liver, spleen, and lung of edited animals. Stars indicate the targeted bases. **c** The predicted editing efficiency statistics based on Sanger sequencing chromatograms from base-edited monkeys by EditR. **d** Base editing efficiency of *RAG1* and *IL2RG* genes assessed by targeted deep sequencing of peripheral blood of surviving mutant monkeys.


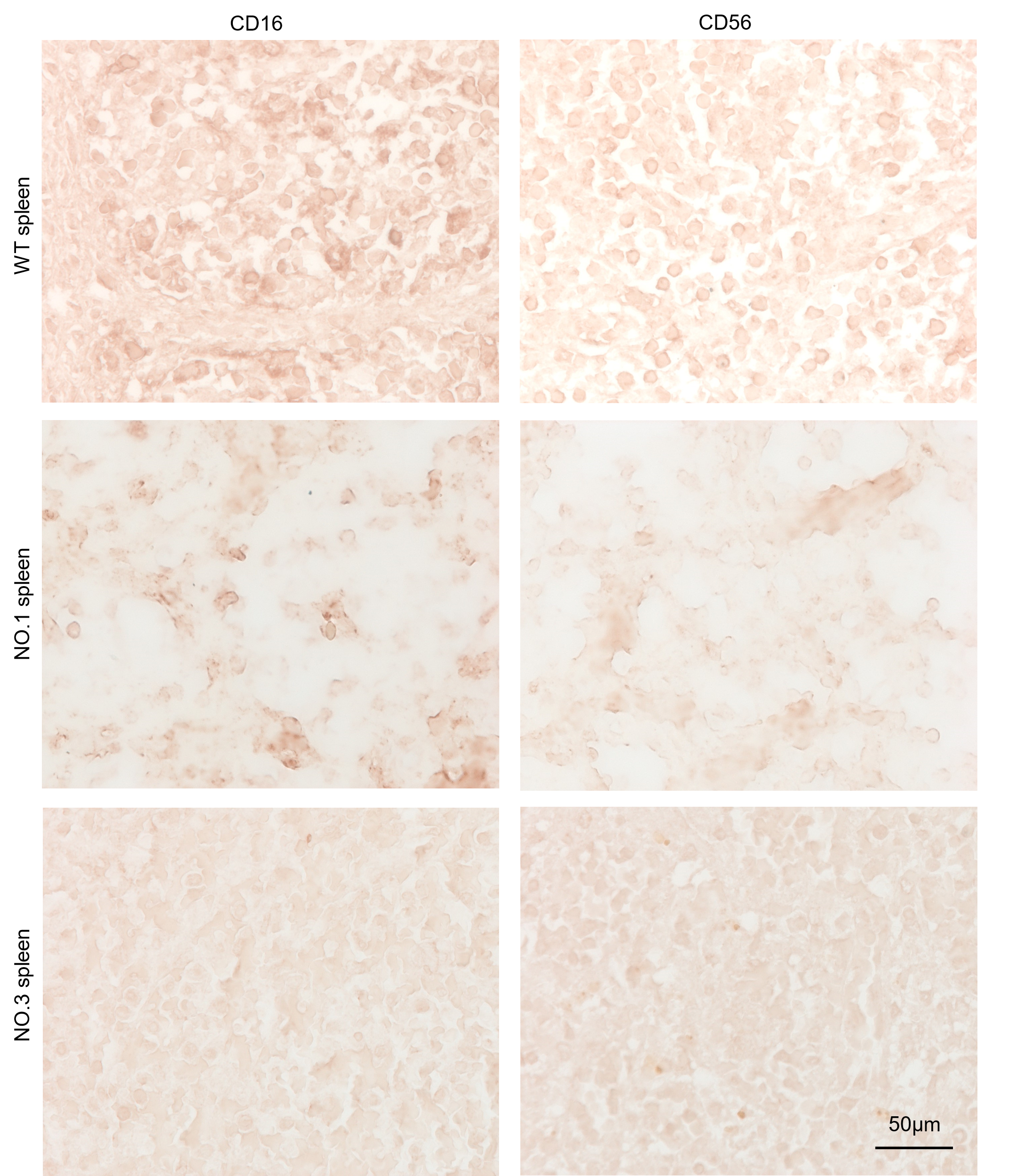


**Fig. S3 Changes of NK cells in the spleen of base-edited monkeys.**


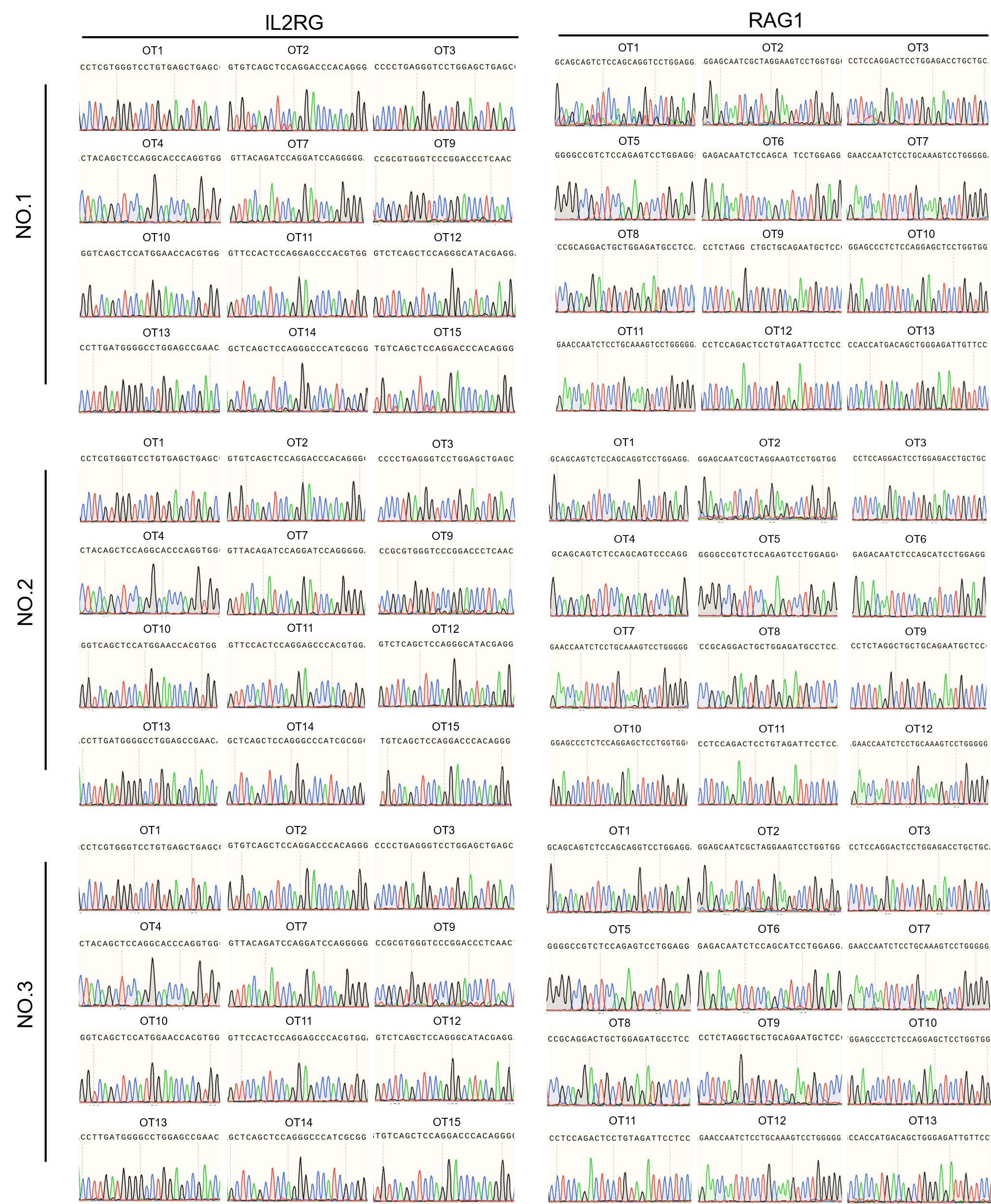


**Fig. S4 Sanger sequencing chromatograms and off-target analysis of base-edited monkeys. Potential off-target sites were predicted using Cas-OFFinder.**


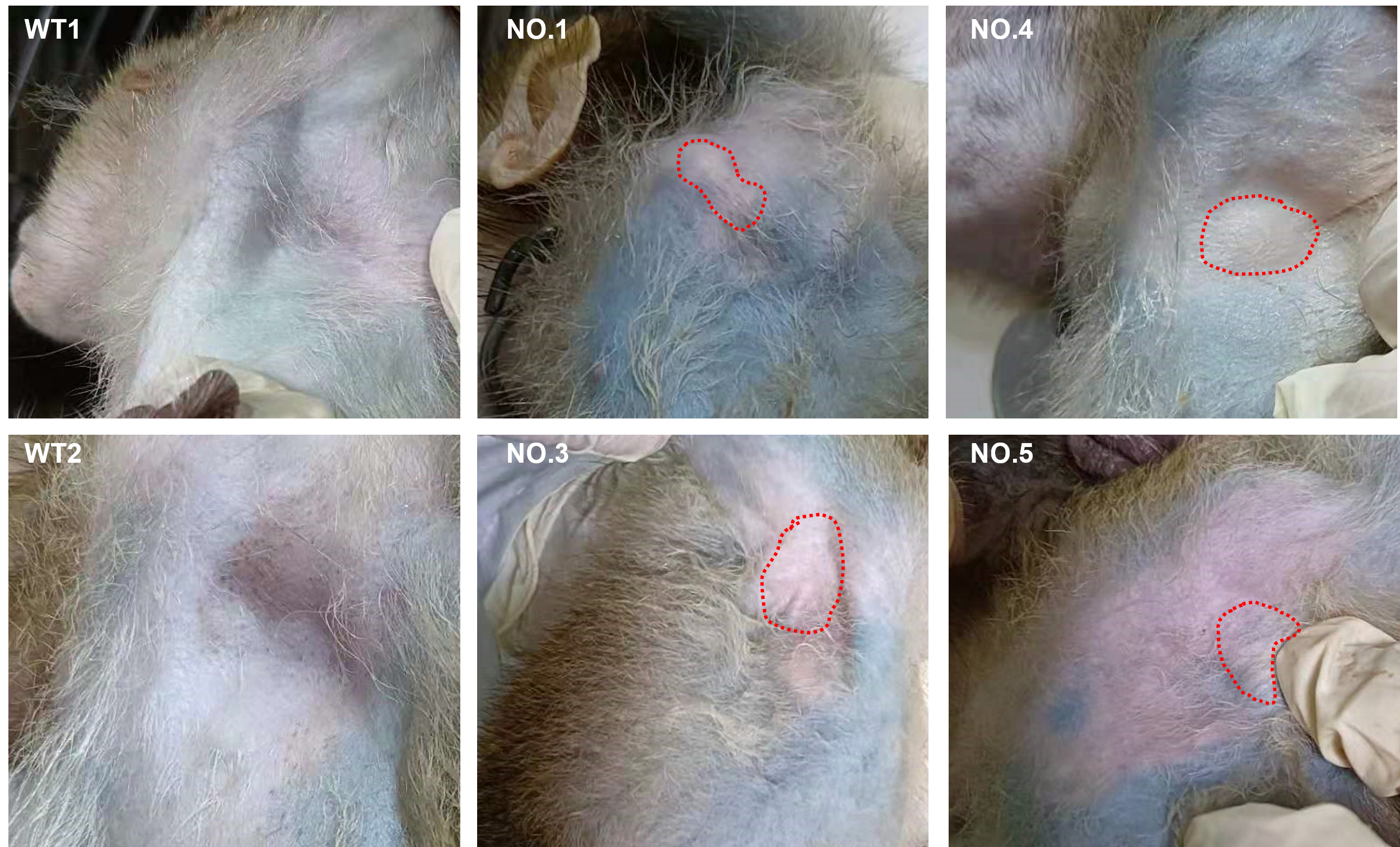


**Fig. S5 Injection of tumor cells in WT and base-edited monkeys.**


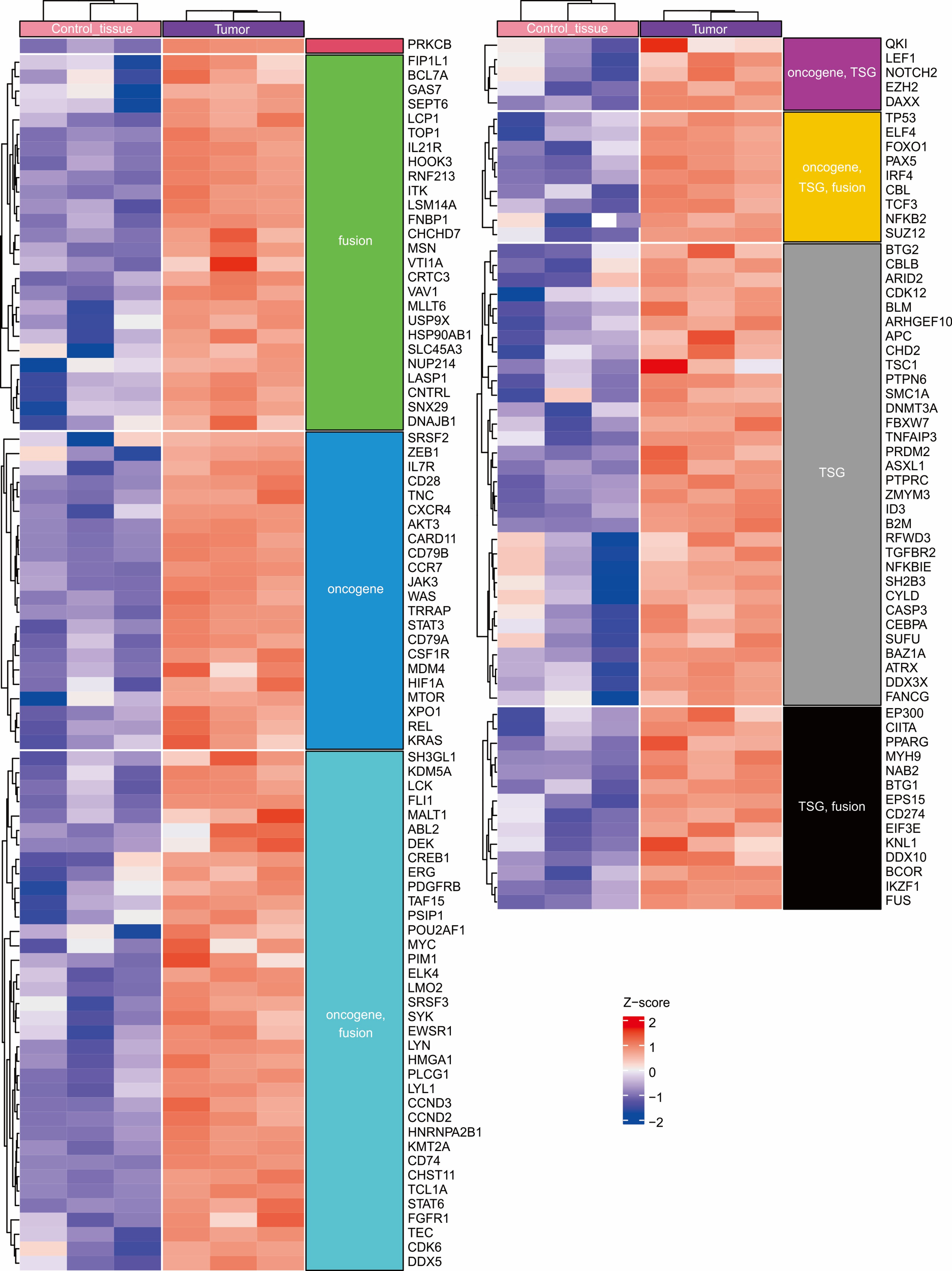


**Fig. S6 Among the tumor-related genes provided by COSMIC Cancer Gene Census, the heat map of highly expressed genes in tumor groups**
